# Marine-Inspired Antimicrobial Peptides Disrupt Gene Expression at the DNA Level

**DOI:** 10.1021/acsinfecdis.5c01000

**Published:** 2025-12-09

**Authors:** Luisa I. Beyer, Johannes Thoma, Leonarda Acha Alarcon, Ivan N. Unksov, Roger Karlsson, Juan S. Inda-Díaz, Alesia A. Tietze

**Affiliations:** † Department of Chemistry and Molecular Biology, Wallenberg Centre for Molecular and Translational Medicine, 3570 University of Gothenburg, Medicinaregatan 7B, 413 90 Gothenburg, Sweden; ‡ Center for Antibiotic Resistance Research in Gothenburg, The University of Gothenburg, Box 100, 405 30 Gothenburg, Sweden; § Department of Infectious Diseases, Institute of Biomedicine, The Sahlgrenska Academy at University of Gothenburg, Box 440, 405 30 Gothenburg, Sweden; ∥ Centre for Microbiome Research, School of Biomedical Sciences, Translational Research Institute, 1969 Queensland University of Technology, 4102 Brisbane, Australia

**Keywords:** antimicrobial peptides, DNA targeting peptides, marine peptides, transcription-translation machinery inhibition, AMPs mode of action

## Abstract

Genome mining of *Streptomyces* sp. H-KF8 combined with sequence engineering yielded two serum-stable, noncytotoxic, nonlytic antimicrobial peptides, L3 and L3-K. Initial studies in uropathogenic *Escherichia coli* suggested membrane effects and nucleoid relaxation, prompting a comprehensive investigation of their mode of action. In this study tandem mass tag (TMT)-based quantitative proteomics revealed extensive proteome remodeling, with 175 and 120 differentially expressed proteins (DEPs) after treatment with L3 and L3-K, respectively. L3 induced predominantly upregulated responses linked to metabolism, RNA processing, transport, and homeostasis, whereas L3-K mainly caused the downregulation of proteins involved in metabolism, transport, and cell structure. Both peptides disrupted ABC transporter-mediated nutrient uptake and elicited stress responses, while L3 specifically perturbed the *mal* regulon, indicative of broader transcriptional dysregulation. Complementary fluorescent dye displacement and in vitro transcription/translation assays demonstrated nonspecific DNA binding, stronger for L3 than L3-K, and potent inhibition of transcriptional and translational processes. Strikingly, inhibitory concentrations paralleled their minimum inhibitory concentrations, directly linking DNA binding and interference with central information processing to antimicrobial activity. These findings reveal that L3 and L3-K primarily act by targeting DNA and interfering with the transcription-translation machinery. Beyond offering mechanistic insights, this study underscores peptides’ potential to act as scaffolds for next-generation antimicrobial peptides with DNA-binding and nonmembrane-lytic activity.

## Graphical abstract

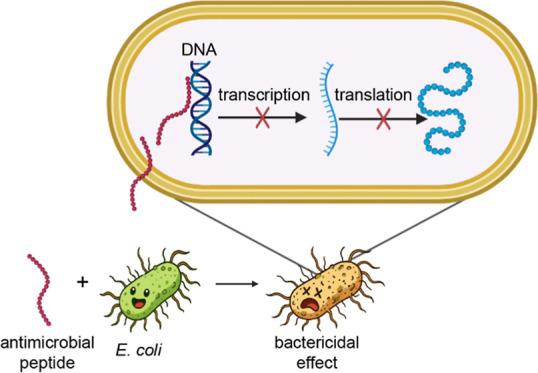

Antibiotic and antimicrobial resistance (AMR) is one of the critical public health challenges nowadays, requiring the urgent need to act against resistant bacteria. As resistance levels rise, medication becomes less effective, making infections increasingly difficult or even impossible to treat.^1^ Developing new compounds to overcome antibiotic resistance mechanisms belongs to one of the strategies to combat AMR. Antimicrobial peptides (AMPs) have beneficial qualities, as they provide complex modes of action potentially, decreasing the risk of developing resistance.^2–4^

In nature, AMPs are produced as secondary metabolites by various organisms to serve as a first-line defense system.^5^ Most AMPs are short, positively charged with an amphiphilic character exhibiting direct microbicidal effect against a wide range of bacteria, fungi, parasites, and viruses.^6–8^ Bacteria can form secondary metabolites known as nonribosomal peptides via a large, multimodular enzyme (nonribosomal peptide synthetase, NRPS). These nonribosomal peptides are structurally diverse, include noncanonical amino acids, and possess various biological activities and pharmacological properties, making them a valuable field of research for novel antimicrobial agents.^9^

AMPs are generally known for their nonspecific membrane-lytic bactericidal activity.^10,11^ Most AMPs target the bacterial membrane by the formation of channels, transmembrane pores, or extensive membrane rupture leading to lysis and direct cell death.^12^ Deeper investigation into the mode of action revealed that not only does the membrane interaction result in bactericidal effects but also intracellular targets can be the leading cause of bacterial cell death. In the case of intracellular targets, cationic AMPs interact with bacterial membranes to translocate into the cytoplasm to access their intracellular site.^3,12,13^ Main intracellular targets are nucleic acid-/protein biosynthesis, protein folding, enzymatic activity, cell division, cell wall biosynthesis, and lipopolysaccharide inhibition.^10,14^ For instance, a tryptophan- and proline-rich peptide, Indolicidin, does permeabilize the bacterial membrane but does not cause cell lysis. Its major mode of action is DNA binding, inhibiting DNA synthesis and inducing filamentation of bacteria.^15,16^ Another example is the proline- and arginine-rich peptide, PR-39 which inhibits protein, RNA, and DNA synthesis, inducing degradation of proteins required for DNA replication.^12,17^ Most DNA-binding AMPs possess nonspecific peptide-phosphate interactions, such as buforin II, a linear α-helical peptide accumulating in the cytoplasm, leading to cell death by minor groove binding to DNA and RNA.^12,18,19^

A deeper understanding of the various modes of action is important to develop more effective and potent AMPs for therapeutic use.^20–23^ The substitution of a single amino acid can drastically alter biological activity, yet our understanding of how the sequence correlates with function remains limited. As a result, the researchers are actively working to establish sequence-activity relationships to guide the design and modification of peptides.^24,25^

In our previous studies, genome mining predictions of strain *Streptomyces* sp. H-KF8 together with sequence engineering led to serum-stable, noncytotoxic qualified hit peptides (L3, L3-K).^26^ Initial mode of action studies in uropathogenic *Escherichia coli* suggest that the peptides potentially act on the cell membrane, allowing the passage of small ions, resulting in the dissipation of the membrane potential. Additionally, the peptides cause relaxation of the nucleoid, which is indicative of DNA packing defects.

In the present study, we aim to gain deeper knowledge about how L3 and L3-K peptides kill bacteria. Tandem mass tag (TMT)-based quantitative proteomics serves as an effective method to elucidate antibacterial mechanisms of drugs against a chosen organism.^27,28^ Differentially expressed proteins (DEPs) of *E. coli* after treatment with L3 and L3-K were identified, and together with gene set enrichment analysis for Gene Ontology (GO) terms and Kyoto Encyclopedia of Genes and Genomes (KEGG) pathways, their biological function and subcellular localization were annotated. Additionally, Protein–Protein Interactions (PPI) were used to understand their complex interactions. Further, the effect on DNA was investigated by fluorescent dye displacement assays and in vitro transcription/translation assays.

## Results and Discussion

Previous studies have shown that the preliminary hit peptide L3 depolarizes the membrane of *E. coli* bacteria but does not rupture the membrane to cause cell lysis. The peptide is positively charged and shows a high sequence similarity to the previously described peptides L3-K, differing from each other by only one C-terminal lysine (Figure 1). Analytical data are reported in (Supporting Information Table S1 and Figures S1 and S2). Both peptides induce relaxation of the nucleoid, indicative of defects in DNA packing, which may result either from their membrane interaction or an independent mechanism. Although sequentially very similar, both peptides showed considerable differences in their bioactivity and inner membrane permeabilization kinetics, as shown by previously reported live-cell microscopy studies.^26^

**Figure 1:**
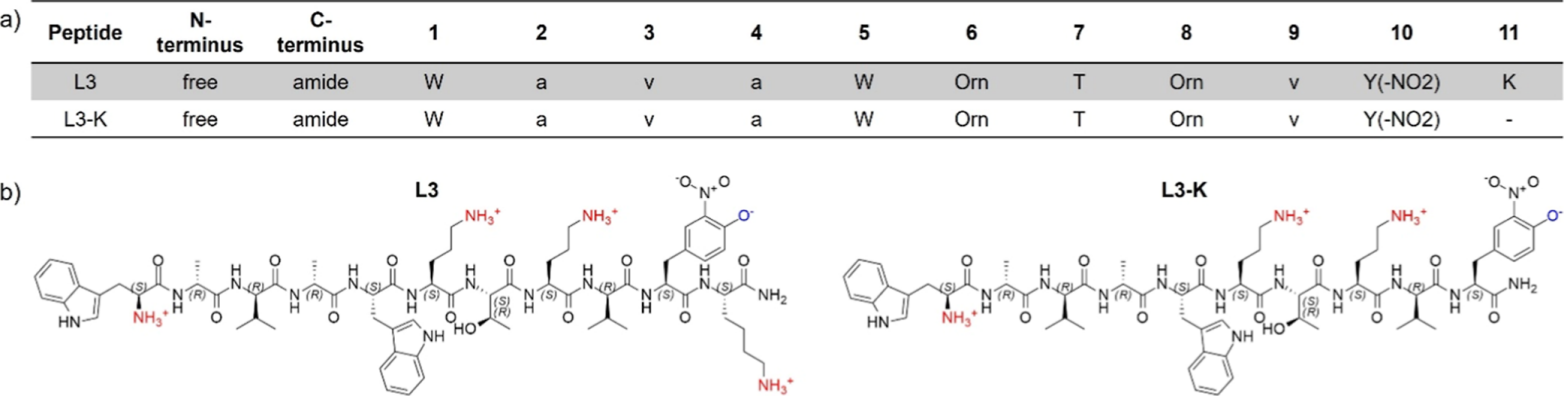
Amino acid composition. (a) sequence and (b) chemical structure of L3 and L3-K. Positively charged residues at physiological pH are shown in red, negatively charged residues in blue, d-amino acids are indicated by lowercase letters, and Y­(-NO_2_) is 3-nitrotyrosine.

Global quantitative proteomics was performed to detect differentially expressed proteins (DEPs) after 10 min of treatment, the time point when different inner membrane permeabilization kinetics were previously observed. With the proteomics study, we focus on developing both a deeper understanding of the peptide-bacterial membrane interactions and investigating how truncation of one amino acid is changing the global protein response of *E. coli* after treatment.

### Quantitative Proteomics Analysis of *E. coli* Upon Treatment with L3 and L3-K

The impact of two AMPs, L3 and L3-K, on global protein expression changes in *E. coli* was investigated by TMT-labeled quantitative proteomics. Differentially expressed proteins (DEPs) between treated bacteria and their control group were identified. The workflow of the quantitative proteomics process is shown in (Figure 2a). Uropathogenic *E. coli* bacteria were treated with 1 × MIC, L3: 45.8 μM; L3-K: 101.0 μM for 10 min of each corresponding peptide. LC–MS/MS measurements after the treatment led to 2602 identified and quantified proteins (∼65% of the proteome) based on the UniProt protein database. Principal component analysis (PCA) and heatmaps were plotted to visualize similarities and clustering between each biological replicate and their conditions (Supporting Information Figures S3–S6). The threshold for identifying DEPs between treated bacteria and their control group was set to a fold change (FC) of ≥/≤1.5 with a p value of ≤0.05. For L3 175 DEPs were identified, with 111 proteins significantly upregulated and 64 downregulated. For L3-K, 120 proteins were quantified, with 38 proteins being upregulated and 82 proteins being downregulated (Figure 2b). The subcellular localization of the DEPs is shown in (Figure 2c,d) and listed together with their FC in (Tables S2 and S3). For L3, the subcellular localization of the proteins is distributed as follows: 2.3% extracellular, 4% outer membrane, 24% periplasm, 18.3% inner membrane, and 51.4% cytoplasm. For L3-K, the distribution is similar: 2.5% extracellular, 5.8% outer membrane, 34.2% periplasm, 15.8% inner membrane, and 41.7% cytoplasm, which means that for both peptides, the most affected proteins are in the periplasm and cytoplasm. The distribution and biological function of quantified proteins will be discussed in detail below.

**Figure 2:**
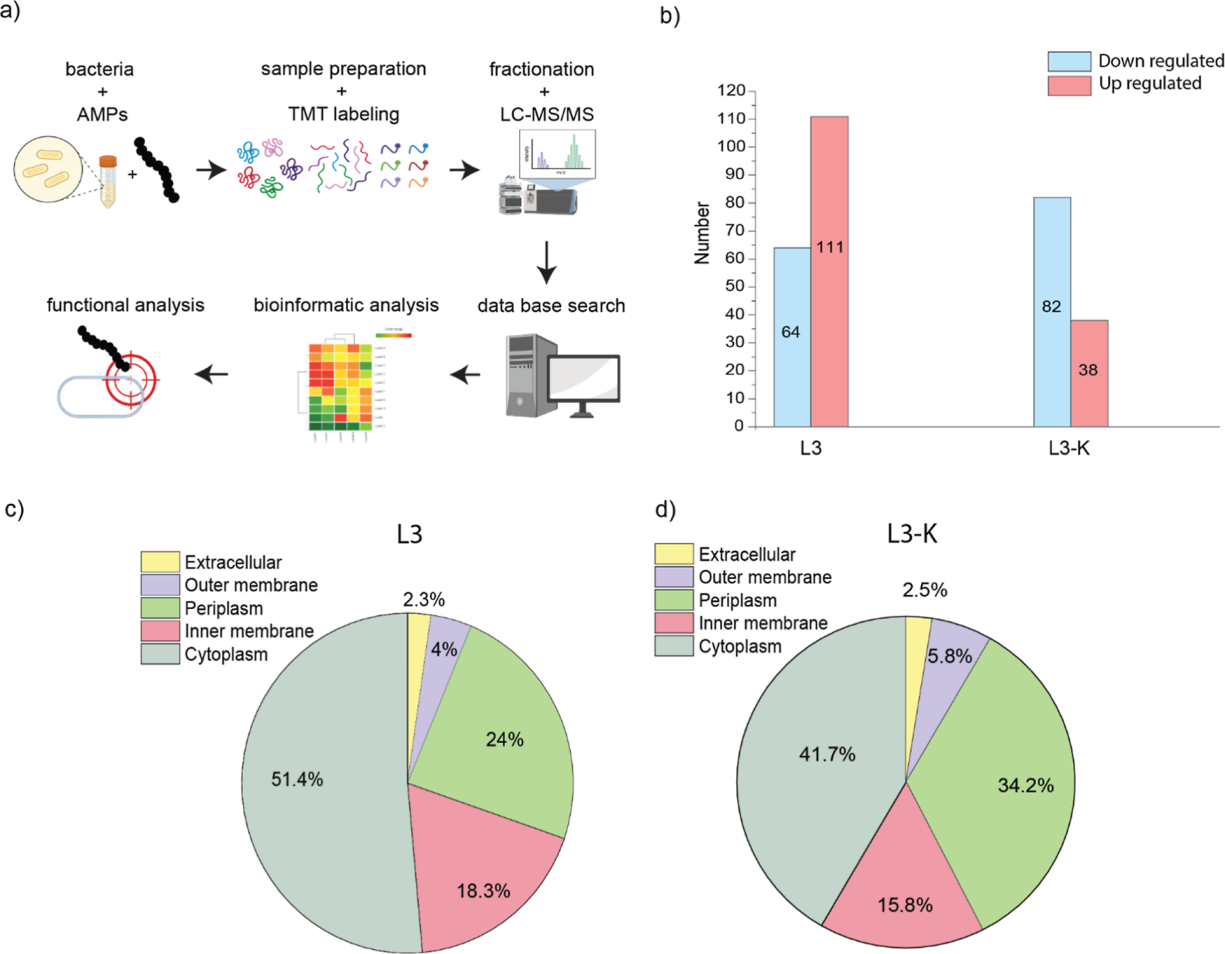
Identification of differentially expressed proteins by *E. coli* upon treatment with AMPs (L3, L3-K) using quantitative TMT-labeled proteomics. (a) Workflow for global proteomics analysis, (b) identification of up- and down-regulated proteins, (c) subcellular localization of DEPs for L3, and (d) subcellular localization of DEPs for L3-K.

The distribution of quantified proteins is illustrated in the volcano plots (Figure 3). The most significantly up- or downregulated proteins, along with their biological functions, are listed in Table 1. For L3 proteins involved in lipid metabolism (FadE and FadB) were significantly downregulated, whereas several proteins with maltose transport (MalM, MalE, MalK, MalF, and LamB) were strongly upregulated, indicating enhanced carbohydrate uptake and utilization. In addition, enzymes related to amino acid degradation (TdcA, TdcB, and TdcD) and biosynthesis (ArgA), as well as carbohydrate acid (IdnD) and nucleobase (PsuG and PreT) metabolism, were significantly upregulated, suggesting a broad metabolic activation in response to the L3 condition. Notably, in the L3-K group, a substantial number of downregulated proteins remain poorly characterized or completely uncharacterized, often labeled as “hypothetical proteins.” These uncharacterized proteins were excluded from further analysis and not listed in the table, and their biological pathways could not be examined. In contrast to L3, the L3-K vs control comparison revealed a marked downregulation of proteins involved in galactose and methyl-galactoside import (MglB and MglC) and their transcriptional repressor (GalS), along with sulfur transferase activity (PspE). Only ArgA, linked to amino acid biosynthesis, was significantly upregulated, suggesting a more restricted metabolic response dominated by repression of carbohydrate transport and sulfur metabolism pathways. To investigate the biological roles of the identified DEPs, bioinformatic analyses were performed using tools that identify groups of genes sharing common biological functions or pathways and determine whether these groups are significantly enriched among the DEPs, instead of evaluating each gene individually.

**Figure 3:**
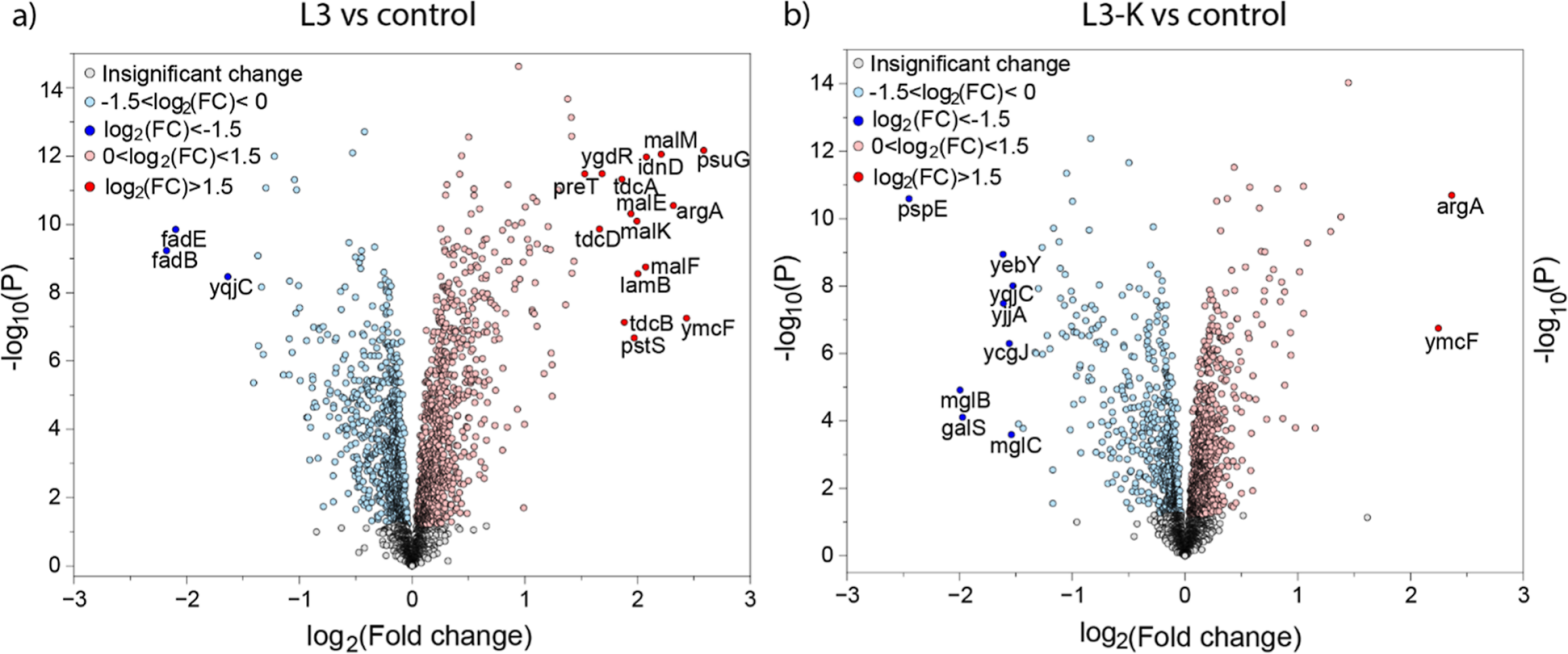
Volcano plots representing up- and down-regulated proteins induced by L3 and L3-K peptides in *E. coli.* The *p*-values and fold changes from the pairwise comparisons between the experimental setups, (a) L3 versus control and (b) L3-K versus control, after empirical Bayes moderation and shrinkage are visualized in the volcano plots. The light red/blue significant up-/down-regulated proteins with an FC of <1.5, and dark red/blue significant up-/down-regulated proteins with a fold change >1.5. The statistical significance is set to a *p*-value of <0.01 to narrow down the search for the most relevant proteins. The gray dots were not found to be significant.

**Table 1:** Most Significant Characterized DEPs with Their Subcellular Localization and Biological Function^a^

gene name	regulation	FC	*p*-value	localization	function
L3 vs Control					
FadE	down	–4,3	4.53 × 10^–7^	inner membrane	Lipid metabolism
FadB	down	–4,7	5.36 × 10^–5^	cytoplasm	Lipid metabolism
MalM	up	4,8	1.65 × 10^–5^	periplasm	maltose transport
MalE	up	4,0	9.04 × 10^–6^	periplasm	maltose transport
MalK	up	4,2	2.28 × 10^–5^	inner membrane	maltose transport
MalF	up	4,5	9.29 × 10^–5^	inner membrane	maltose transport
LamB	up	4,3	1.30 × 10^–4^	outer membrane	maltose transport
IdnD	up	4,3	7.19 × 10^–7^	cytoplasm	carbohydrate acid metabolism
PsuG	up	6,3	2.29 × 10^–6^	cytoplasm	nucleobase catabolic process
PreT	up	3,0	1.41 × 10^–5^	cytoplasm	pyrimidine base degradation
TdcA	up	3,8	2.58 × 10^–5^	cytoplasm	amino acid degradation
ArgA	up	5,1	5.89 × 10^–6^	cytoplasm	Amino acid biosynthesis
TdcD	up	3,3	2.00 × 10^–5^	cytoplasm	amino acid degradation
TdcB	up	4,1	9.58 × 10^–4^	cytoplasm	amino acid degradation
PstS	up	4,1	8.11 × 10^–5^	periplasm	phosphate ABC transporter
L3-K vs Control					
PspE	down	–5,2	1.57 × 10^–5^	periplasm	sulfur transferase
MglB	down	–4,4	2.31 × 10^–3^	periplasm	galactose/methyl galactoside import
GalS	down	–4,0	3143 × 10^–3^	cytoplasm	repressor of the mgl operon
MglC	down	–3,2	9.72 × 10^–3^	Inner membrane	galactose/methyl galactoside import
ArgA	up	5,4	2.87 × 10^–5^	cytoplasm	amino-acid biosynthesis

a Biological functions are based on the UniProt database.

### Bioinformatic Pathway Analysis of Differentially Expressed Proteins

To analyze functional roles of proteins, two widely used databases, GO and Kyoto Encyclopedia of Genes (KEGG), were used to connect proteins to enriched biological terms and pathways (Figures S7 and S4). GSEA was performed to identify enriched genes in a ranked list, established on the normalized enrichment score (NES). NES is based on the normalized expression change to limit differences in gene set sizes, enabling direct comparison across all gene sets. Statistical significance is assessed using an adjusted p-value with false discovery rate (FDR) applied to control for multiple testing and limit the occurrence of false positives.

GO annotation identified the functions of DEPs through standardized biological terms (S7). GO enrichment showed that in the L3 condition, DEPs were mainly linked to metabolism, RNA processing, transport, homeostasis, and cell development. Downregulation of lipid degradation pathways (fatty acid oxidation and catabolism) indicated reduced energy production and a stress-induced metabolic shift, while decreased rRNA methylation suggested energy conservation. In L3-K, similar GO terms were enriched but focused on amino acid metabolism, biosynthesis, and transport. Most DEPs were upregulated, except those related to carbohydrates and import into cells, reflecting stress adaptation and a shift from glucose to alternative carbon sources. KEGG pathway analysis supported these results, in L3, carbohydrate metabolism (pentose and glucuronate interconversions) was upregulated, whereas translation and ribosome assembly were downregulated, indicating an increased energy demand and reduced protein synthesis. In L3-K, downregulation of energy metabolism pathways, including ABC transporters and sulfur metabolism, aligned with known antibiotic stress responses in *E. coli*,^29^ while arginine biosynthesis was specifically upregulated (Figure 4).

**Figure 4:**
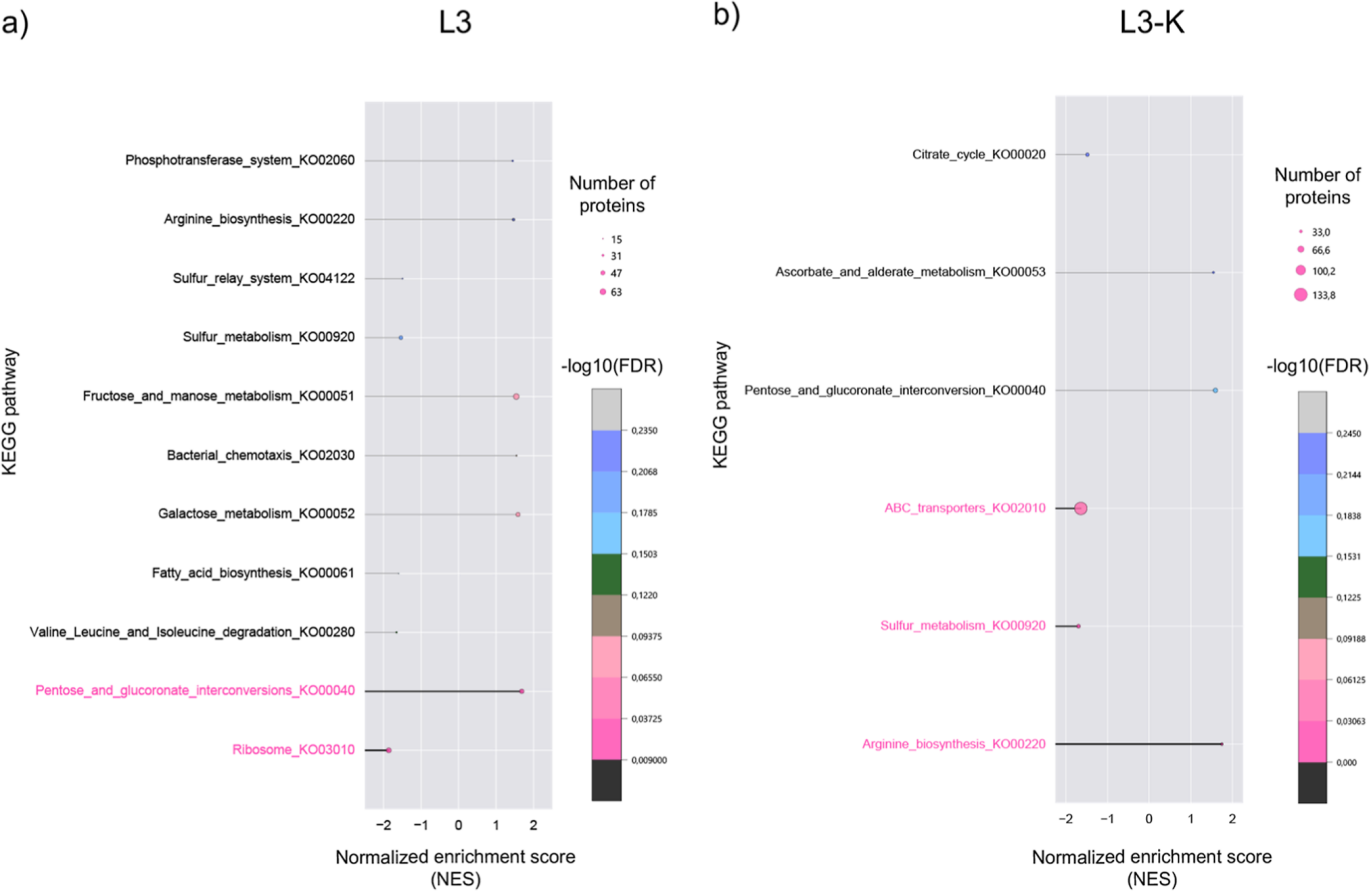
GSEA of KEGG pathways. (a) KEGG pathways for L3; pink colored with an FDR value of ≤0.05; (b) KEGG pathways for L3-K; pink colored with an FDR value of ≤0.05.

PPI analysis using the STRING database linked DEPs to enriched biological processes and pathways. Networks were constructed for 175 DEPs in L3 and 120 in L3-K (Figures S8 and S9). In L3, the main affected processes involved transport and metabolism, including clusters related to arginine biosynthesis, catabolic reactions, and nucleobase metabolism. In L3-K, similar clusters were observed, particularly involving metabolic pathways, ABC transporters, sulfur metabolism, arginine biosynthesis, and siderophore production. Downregulation of siderophore-associated proteins (EntB, EntC, EntE, and EntH) suggested reduced enterobactin synthesis, a stress response mechanism limiting iron uptake and limiting reactive oxygen species damage.^30^ The siderophore cluster’s connection to FepB, an ABC transporter protein for ferric-enterobactin uptake, further supports peptide-mediated modulation of ABC transport systems (Tables S4 and S5).^31^

Both peptides affected multiple ABC transporters, integral membrane proteins that actively transport diverse substrates across the bacterial membrane.^31,32^ In *E. coli*, substrate passage typically involves outer membrane porins followed by ABC transporter-mediated import or efflux across the inner membrane. Although affected transporters differed between L3 and L3-K, all belonged to substrate importer families.

In L3, ABC transporters related to carbohydrates (Mal and Rbs), peptide (Dpp), and phosphate (Pst and Ugp) import were upregulated, whereas sulfur (Ssu and Tau) and iron (Fep) transporters were downregulated. The maltose transporter MalFGK_2_ showed the strongest upregulation (MalF FC 4.5; MalK FC 4.2), accompanied by LamB porin (FC 4.3) and transcriptional activator MalT (FC 1.7), indicating activation of the maltose regulon under glucose limitation. However, LamB’s substrate selectivity makes it unlikely to serve as an entry pathway for L3.^33^ DppD (FC 2.0), a nucleotide-binding domain of the dipeptide transporter, along with DppA and DppB, was also upregulated, suggesting potential roles in peptide import.^34,35^

In contrast, nine of ten ABC transporters in L3-K were downregulated, notably the galactose transporter Mgl (MglA FC—3.0, MglB FC—4.4, MglC FC—3.2). Only the phosphate-binding protein PstS was upregulated under both conditions (FC 4.1 and 2.4). These results indicate that both peptides modulate ABC transporter expression, likely influencing membrane transport or permeability rather than forming transmembrane pores.

Given that several affected transporters (e.g., FepB and PstS (Tables S4 and S5)) are associated with DNA damage response pathways, fluorescence displacement assays were conducted to assess peptide–DNA interactions.^36^ Additionally, effects on the maltose regulon, which mediates maltose and malto-oligosaccharide uptake, were further investigated.

### *mal* Regulon is Not Directly Activated by L3 and L3-K

Several of the most pronounced DEPs identified in the proteomics analysis are components of the *mal* regulon, which mediates the uptake and metabolism of maltose and malto-oligosaccharides in *E. coli*. In light of the tight regulation by catabolite repression via cAMP-CRP, as well as by the MalT transcriptional activator, the prominence of *mal* regulon proteins among DEPs raises several mechanistic and regulatory questions. Notably, cells used for proteomic analysis were sampled after 10 min of exposure to the AMPs L3 or L3-K at MIC. Given the short duration of exposure to the peptides, such a rapid and robust upregulation of the mal genes is unexpected.^37^ Therefore, observing such a dramatic change in expression within 10 min suggests either a rapid regulatory disturbance or a breakdown of normal repression. Moreover, the cells were grown in 10% brain heart infusion (BHI), a dilute complex medium that contains glucose. Under these conditions, catabolite repression is expected to suppress expression of the *mal* regulon, as upstream regulatory regions of the *mal* operons harbor several catabolite activator protein binding sites.^33^ The *mal* regulon is known to become derepressed as part of the cellular response to carbon starvation,^38^ potentially permitting its further induction in response to a suitable stimulus.

To determine whether the observed up-regulation of *mal* genes was a direct or indirect effect of AMP exposure, a transcriptional reporter system was constructed. The regulatory region of the *malK*–*lamB*–*malM* operon was cloned upstream of an sfGFP coding sequence in a low-copy plasmid and transformed into the *E. coli* K-12 reference strain MG1655. These bacteria were grown under the same conditions as in the previous proteomics experiments and exposed to the AMPs L3 or L3-K, the control peptide GGH, or grown in plain 10% BHI as well as 10% BHI supplemented with maltose as negative and positive controls, respectively. Cultures were incubated at 37 °C, while both optical density and GFP fluorescence were monitored over a period of 180 min. Growth curves showed that AMP-treated cultures exhibited only a minor initial increase in optical density, which plateaued after approximately 45 min (Figure 5a), confirming growth arrest or onset of cell death as a consequence of the antimicrobial activity of both peptides. In contrast, GGH had no impact on growth and showed normal growth trajectories comparable to untreated controls and maltose-supplemented cultures. At the start of the experiment, all cultures displayed a relatively low GFP signal, which remained almost constant over the first 80 min (Figure 5b). In both AMP-treated cultures GFP fluorescence remained low for the remainder of the experiment, which is in agreement with the growth arrest observed under these conditions. In contrast, a marked increase in GFP fluorescence was observed in the remaining cultures beginning around 90 min and reaching a maximum at approximately 120–150 min, which was further pronounced in the presence of maltose, which is consistent with canonical induction of the *mal* regulon due to glucose depletion as well as maltose-dependent activation of MalT.^39^

**Figure 5:**
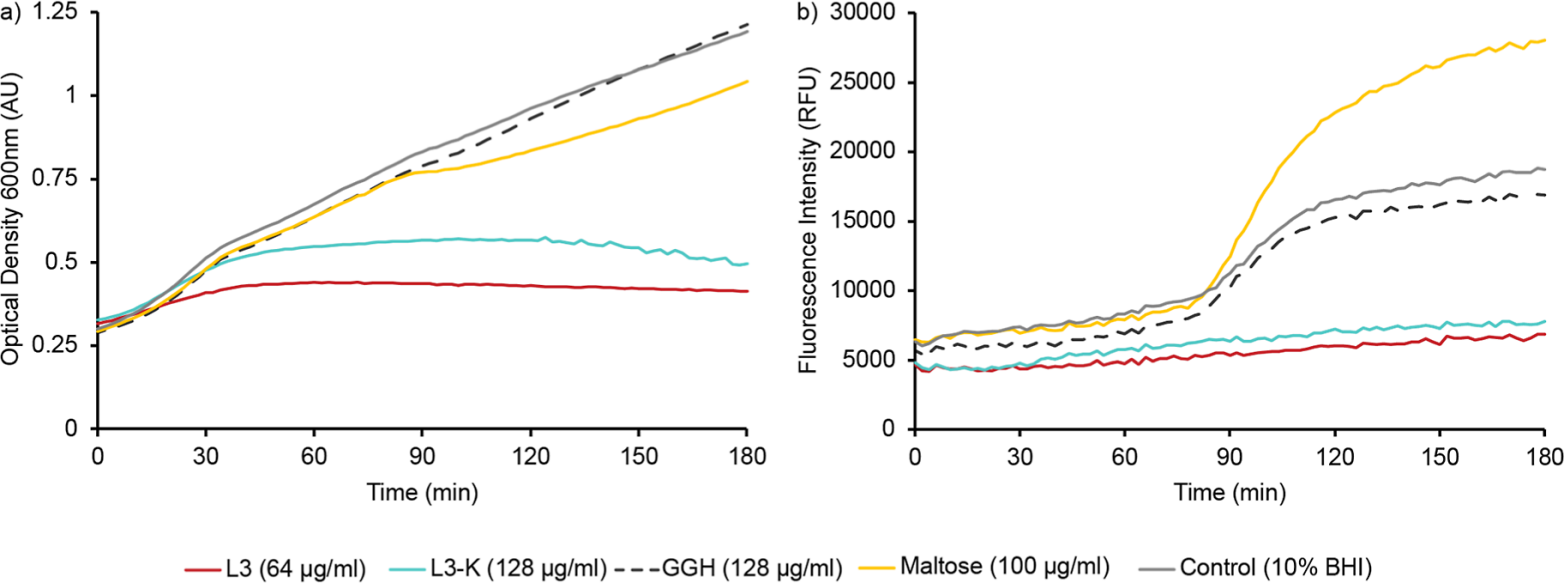
Activation of the *mal* regulon. (a) Time course of cell density of *E. coli* MG1655 carrying a transcriptional fusion of the *malK* regulatory region to GFP grown in 10% BHI medium in the presence and absence of L3, L3K, or maltose. (b) Time course of GFP fluorescence of the cultures shown in (a) All traces represent averages of 3 independent measurements.

The results indicate that the AMPs do not directly activate transcription of the *malK*–*lamB*–*malM* operon. The apparent upregulation of *mal* regulon members in the proteomic analysis may therefore be explained as a consequence of an untargeted regulatory disruption in AMP-treated cells compared to untreated cells. While *mal* expression is expected to be strongly repressed due to glucose-dependent catabolite repression, due to impaired gene regulation under stress by the AMPs, the *mal* regulon may therefore appear relatively derepressed compared to untreated bacteria, giving rise to the observed differential expression profile. When combined with the prior observation that AMPs cause relaxation of the bacterial nucleoid, this is indicative of strong DNA interactions. The reporter assay therefore casts the proteomic data in a new light, suggesting that the observed *mal* deregulation reflects a broader disturbance of transcriptional control rather than a targeted effect on specific regulatory pathways. This, in turn, supports the hypothesis that the peptides may exert their antimicrobial activity by inducing a global regulatory collapse at the DNA level.

### Peptide–DNA Interaction

To further investigate the DNA-peptide interactions of L3 and L3-K, we measured the displacement of two standard DNA-bound fluorophores. DAPI binds the minor groove and prefers AT-rich DNA at low dye/phosphate ratios.^40,41^ Ethidium bromide (EtBr) binds mainly by intercalation and can also associate electrostatically with the DNA surface.^42–45^ We assessed displacement on three supercoiled plasmids of different sizes and sequences (pUC19, pSY97P, and pY359). Supercoiled plasmids were chosen over short oligonucleotides to retain physiological topology and structural heterogeneity that influence the binding mode. The fluorescence of free dyes was low compared to when bound to DNA, and it is not affected by either peptide (Figure S8). Therefore, a decrease in fluorescence indicates a loss of dye from DNA.

To benchmark the assay, we included two references with known modes of interaction with DNA. The first is positive control daunomycin, which is an intercalator with additional minor-groove contacts via its amino sugar.^46^ The second reference is Gly-Gly-His (GGH) amino-terminal Cu­(II) and Ni­(II) (ATCUN) binding peptide, which is a negative control for intercalation and is a minor groove binder.^47^ As expected, daunomycin efficiently displaced EtBr at a low drug/phosphate ratio and also reduced DAPI fluorescence. Whereas the ATCUN peptide caused a minimal displacement of EtBr and the modest effect on DAPI (Figure S10). These controls validate that the assay distinguishes intercalation from groove binding.

Relative to controls, L3 and L3-K displaced DAPI at slightly lower peptide/phosphate ratios than EtBr across all plasmids (Figures 6, S9 and S10), placing their behavior between the ATCUN and daunomycin. This trend supports a two-step binding model in which initial minor-groove association is followed by partial progression toward intercalation at higher peptide/phosphate ratios.^46^ L3-K required higher ratios than L3 to reach comparable displacement of both dyes, indicating a weaker overall DNA affinity.

**Figure 6:**
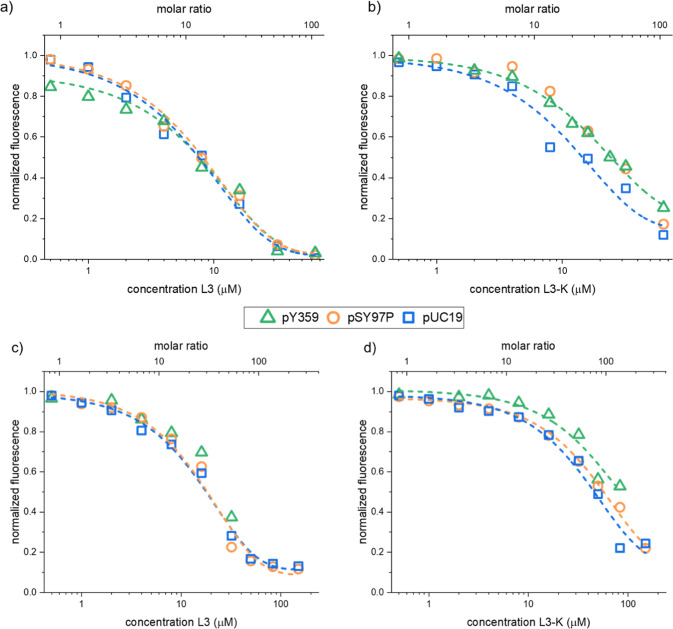
Displacement of (a,b) DAPI and (c,d) ethidium bromide from supercoiled plasmid DNA by (a,c) L3 and (b,d) L3-K. Peak fluorescence (460 nm for DAPI, 610 nm for EtBr) is normalized to the value before peptide addition (raw traces in Figure S9). Lower fluorescence indicates dye displacement. Molar ratio is peptide concentration relative to DNA phosphate concentration. Plasmids: pUC19—blue squares, pSY97P—orange circles, and pY359—green triangles. Dashed lines are exponential fits. Assay controls are shown in Figure S10.

The observed differences can be rationalized by the structural features of the peptides. Aromatic side chains (Trp, Y-NO_2_) favor π–π stacking with base pairs, enabling intercalation once the peptide is localized at the duplex. Cationic residues (Orn and Lys) provide electrostatic attraction and hydrogen bonding to the phosphate backbone and can stabilize groove-bound states, while backbone flexibility of linear peptides allows conformational adaptation from groove recognition to partial insertion. The reduced potency of L3-K can be explained by the loss of the terminal cationic group (Lys) compared to L3, which decreases electrostatic interaction with DNA and disfavors intercalation.

Overall, the similar behavior across plasmids indicates that binding is largely sequence-dependent and governed by general duplex features (groove accessibility and base-stacking sites), which is in line with prior observations for aromatic, cationic DNA-binding peptides reported in the literature.^48^

### L3 and L3-K Disrupt Bacterial Transcription/Translation

The results presented to this point suggest that the AMPs L3 and L3-K induce a widespread dysregulation of gene expression in *E. coli* by nonspecifically binding to DNA, suggesting that their antimicrobial activity may stem from interference with the bacterial transcriptional/translational machinery. To further evaluate the functional DNA binding consequences, we devised a cell-free in vitro transcription–translation (IVT) system. Thereby, the use of an IVT allows the characterization of gene expression detached from the complexity of cellular metabolism, cell cycle regulation, and cell viability.^49^ To this end we first employed an *E. coli*-based cell extract utilizing the pET expression system^50^ to produce sfGFP from a plasmid template, a system which serves as a robust and widely used benchmark for assessing IVT efficiency.^51,52^ In the presence of either L3 or L3-K, GFP production was drastically reduced compared to that of untreated controls, indicating a strong inhibitory effect on the transcription and/or translation processes (Figure 7a). Consistent with prior proteomics findings, L3 had a slightly stronger inhibitory effect than L3-K. In contrast, the control peptide GGH, which lacks antimicrobial activity and does not bind DNA, had no measurable effect on GFP expression.

**Figure 7:**
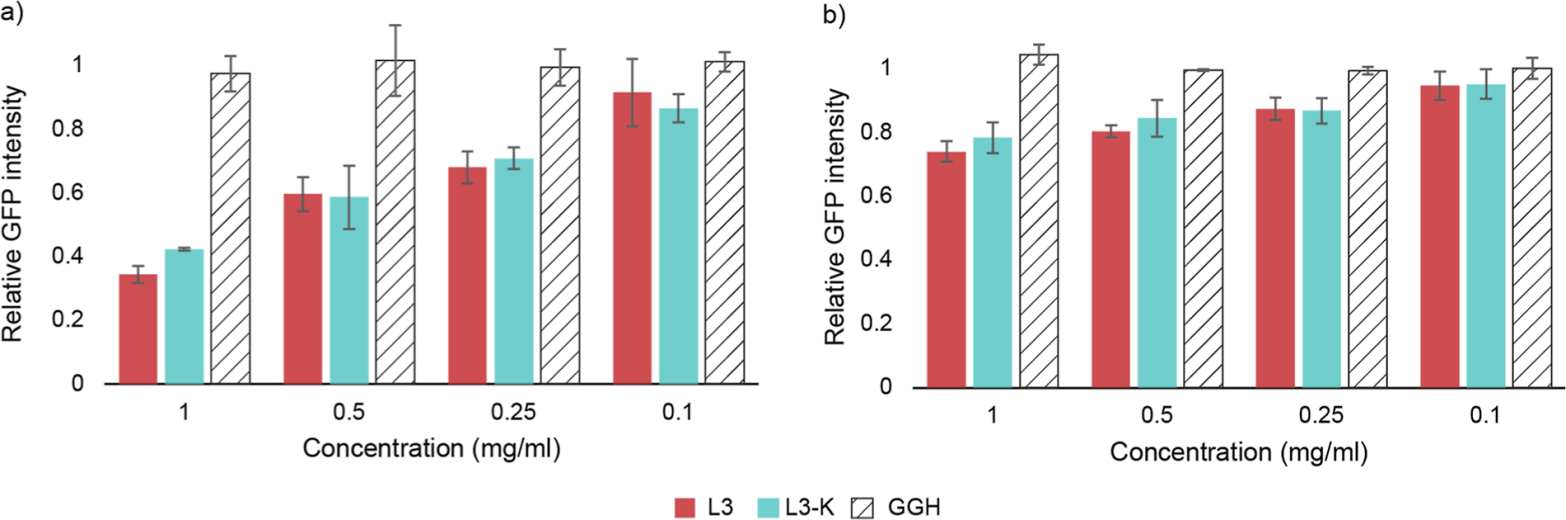
Normalized GFP intensity determined in the presence or absence of different concentrations of L3 (717, 358, 179, and 72 μM), L3-K (789, 395, 197, and 79 μM), or GGH using (a) a T7 RNA polymerase-based or (b) an endogenous bacterial RNA polymerase-based in vitro transcription/translation system. All measurements were normalized to a peptide-free control. Bars represent averages of 3 independent measurements; error bars show standard deviation.

One potential limitation in determining the inhibition of the transcription machinery using IVT systems optimized for high-level protein production is their inherent reliance on the bacteriophage-derived T7 RNA polymerase. These systems are routinely prepared based on extracts from cells overproducing the T7 RNA polymerase and consequently contain very high levels of the enzyme. To evaluate whether the same inhibitory effect can be observed on the endogenous bacterial RNA polymerase from *E. coli*, we repeated the IVT assay using cell extract from *E. coli* not expressing the T7 RNA polymerase to produce sfGFP from a plasmid template under control of the constitutive *pBla* promoter. Again, we observed a reduction in GFP production in the presence of either L3 or L3-K (Figure 7b), despite it being less pronounced when compared to the previous experiment utilizing the T7 RNA polymerase. Due to the differences in experimental parameters—including RNA polymerase concentration, promoter strength, and translation initiation efficiency of the constructs, resulting in generally lower expression levels using endogenous promoters;^53^ a direct quantitative comparison between the two experiments is not meaningful. Nevertheless, both experiments consistently show the same trend, i.e. a reduction of GFP fluorescence as the concentration of either L3 or L3-K increases.

In both experiments, the concentration range at which inhibition occurred was in the same range as the MIC values previously determined in cellular assays, further supporting that impairing the transcription/translation machinery through DNA interactions lies at the core of the peptides’ antimicrobial activity. Such a mechanism of action aligns well with previous growth data, which showed that AMP-treated bacteria remained viable or metabolically active for approximately 45 min, suggesting that while the existing proteome remains functional during this window, it is no longer replenished due to a collapse of controlled gene expression. However, although not directly tested here, it is also plausible that DNA replication is similarly impaired under these conditions.

## Conclusion

TMT-based quantitative proteomics revealed that treatment of *E. coli* with the AMPs L3 and L3-K results in widespread proteomic changes with 175 and 120 DEPS, respectively. L3 induced a predominantly upregulated response, while L3-K caused more downregulation, though both peptides strongly affected proteins associated with stress response. Functional enrichment analysis indicated that L3 primarily impacts metabolism, RNA processing, transport, homeostasis, and cell development, with KEGG pathway analysis suggesting increased energy demand accompanied by downregulation of ribosome assembly and protein synthesis. In contrast, L3-K primarily influenced proteins linked to metabolism, transport, and cell structure, with downregulation pointing to perturbations in energy metabolism. Both peptides affected ABC transporters, specific substrate importers, highlighting alterations in nutrient uptake, including connections to bacterial DNA damage responses.

Further, L3 disrupted the *mal* regulon, normally controlling maltose and malto-oligosaccharide metabolism, which, together with reporter assays, confirms the assumption of a broader disturbance of transcriptional control rather than a pathway-specific effect. Complementary fluorescent dye displacement assays demonstrate nonspecific DNA binding, with indicated intercalation and minor groove involvement. The binding is stronger for L3 than for L3-K, which suggests that lysine facilitates the interaction with DNA. Cell-free IVT assays confirmed a potent inhibition of transcription and/or translation, with L3 exerting slightly stronger effects than L3-K. Importantly, the inhibitory concentrations closely matched previously determined MIC values, directly linking DNA binding and interference with transcriptional/translational machinery to the antimicrobial mechanism of these peptides.

Together, these findings identify DNA-targeting and the disruption of central information processing pathways as the primary antimicrobial mode of action for L3 and L3-K. This work not only elucidates the molecular basis of their activity but also highlights their potential as templates for the design of next-generation, nonmembrane-lytic AMPs with DNA-binding properties.

## Methods

### Peptide Synthesis, Purification, and Characterization

L3 and L3-K were synthesized by automated Fmoc-based SPPS on an INTAVIS MultiPep synthesizer using HBTU and NMM as reported by Beyer et al.^26^ In short, L3 and L3-K peptides were synthesized on Rink amide resin and GGH ATCUN was synthesized using chlorotrityl chloride resin. Global deprotection and cleavage was accomplished in a mixture of 92.5% trifluoroacetic acid (TFA), 5% water, and 2.5% TIPS. Crude and purified peptides were analyzed by analytical RP-HPLC on a Waters e2695 Alliance system (Waters, Milford, MA, USA) employing a Waters 2998 photodiode array (PDA) detector equipped with an ISAspher Xela 100-1.7 C18 column (50 × 2.1 mm). HPLC eluent A was water (0.1% TFA), and eluent B was acetonitrile (0.1% TFA) (detection at 214 nm). Preparative scale purification of the peptides was achieved employing a Waters 1525 binary gradient pump and a Waters 2998 PDA detector with HPLC eluent A water (0.1% TFA) and eluent B acetonitrile (0.1% TFA). The molecular weight of the purified compounds was confirmed by ESI mass spectrometry on a Waters SYNAPT G2-Si HD-MS spectrometer equipped with a Waters Acquity UPLC system (Waters, Milford, MA, USA). Leu-enkephalin was used as a reference compound for high-resolution measurements. Chromatograms are reported in Supporting Information (Table S1, Figures S1, S2, and S11).

### Bacterial Strain and Growth Conditions

*E. coli* strain CCUG 31246 was received from the Culture Collection of the University of Gothenburg (CCUG) and was cultured in 1:10 brain heart infusion (BHI) medium at 37 °C overnight with continuous shaking at 180 rpm.

### Sample Preparation for Quantitative Proteomics

The preinoculum of *E. coli* CCUG 31246 was diluted and grown until it reached a concentration of 2.4 × 10^8^ cells/mL (OD = 0.3). Subsequently, 5 mL of bacterial suspension without peptide, 5 mL of bacterial suspension with peptide L3 at 1× MIC (64 μg/mL), and 5 mL of bacterial suspension with peptide L3-K at 1× MIC (128 μg/mL) treatment were incubated in a temperature-controlled shaker (37 °C, 180 rpm) for 10 min. Each treatment group had 6 biological replicates. Afterward, the bacterial biomass was collected by centrifugation (4000*g*, 4 °C, 8 min). The collected pellets were resuspended and washed 3× with 1 mL of PBS 1× and centrifuged at (4000*g*, 4 °C, 8 min). The cell precipitate was adjusted to OD = 1.5 in 1.5 mL of PBS 1×, collected by centrifugation, as described above, and stored at −20 °C.

### Relative Quantification by Proteomics

Details of the experiment are provided in Supporting Information. (Relative quantification by proteomics). Proteins were extracted in SDS buffer, and protein concentrations were determined using BCA. Samples were processed using a modified SP3 method. Briefly, samples (50 μg) were reduced (100 mM DTT) and alkylated (20 mM iodoacetamide). Proteins were precipitated on the Sera-Mag SpeedBeads (Cytiva) by acetonitrile, washed, and dried at room temperature. Beads were resuspended in 100 mM HEPES, and proteins were digested with trypsin/Lys-C mix [1:25] for 2 h and trypsin [1:50] overnight. Beads were removed, and peptides were labeled using TMTpro 18-plex isobaric mass tagging reagents (Thermo Fisher Scientific) according to the manufacturer’s instructions. The labeled samples were pooled and purified by HiPPR Detergent Removal Resin and Pierce and Peptide Desalting Spin Columns (both Thermo Fisher Scientific). The TMT-set was fractionated using basic reversed-phase chromatography (bRP-LC, pH10) over 70 min into 24 fractions. Each fraction was analyzed on an Orbitrap Eclipse Tribrid mass spectrometer equipped with the FAIMS Pro ion mobility system interfaced with an nLC 1200 liquid chromatography system (all Thermo Fisher Scientific). Peptides were separated on a C18 40 cm column over 90 min, and data were acquired with SPS MS3 method. Raw files were processed and analyzed with Proteome Discoverer (ver 3.0, Thermo Fisher Scientific) by matching against the UniProt *E. coli* strain K-12 database (downloaded 2023/12/06) using Sequest as a search engine.

The MS proteomics data has been deposited to the ProteomeXchange Consortium (http://proteomecentral.proteomexchange.org) via the PRIDE partner repository^54^ with the data set identifier PXD068455.

### Statistical Analysis

Fold changes were calculated by comparing treated bacteria with their untreated controls. The significance of the DEPs was calculated based on a *t*-test of the log_2_-transformed data. Proteins were defined as differentially expressed with a fold change of ≥1.5 or ≤ −1.5 and a *p*-value of ≤0.05.

The protein signals were analyzed separately as log_2_-transformed, and using log_2_ relative abundances as explained in.^55,56^ Differential protein abundance analysis on the log_2_-transformed and log_2_ relative abundances was performed using the Limma and DEqMS packages in R. Experimental conditions were encoded as a factor with three groups (Control, L3, and L3K, each with *n* = 6 replicates). A design matrix without an intercept was constructed to model group-specific effects. A linear model was fitted to the normalized protein intensity matrix, generating coefficients corresponding to each experimental condition. To evaluate pairwise contrasts of interest, we defined three comparisons: L3 versus Control, L3-K versus Control, and L3 versus L3-K. Contrasts were specified and applied to the linear model, followed by empirical Bayes variance moderation to stabilize inference across proteins. Variance estimates were further refined using peptide-spectrum-count information to improve the accuracy of differential abundance testing.

To visualize the overall variation in protein abundance profiles, we performed principal component analysis (PCA) on the log_2_-transformed and log_2_ relative abundances. PCA was conducted without variance scaling. Sample separation along the first two principal components (PC1 and PC2) was visualized and colored according to the experimental group (Figures S3 and S4). Heatmaps were generated to visualize relative protein abundance patterns across the samples. The log_2_-transformed and log_2_ relative abundances were plotted using the heatmap.2 function in R. Sample grouping indicated experimental conditions (Figures S5 and S6). The *p*-values and fold changes from the pairwise comparisons between the experimental setups after empirical Bayes moderation and shrinkage are visualized in the volcano plots (Figure S7). Volcano plots were visualized by using OriginPro 2025.

### Bioinformatical Analysis and Gene Set Enrichment

The subcellular localization for all DEPs was performed with DeepLocPro 1.0.^57^ Their Protein–protein Interactions were analyzed with STRINGv.12 ^58,59^ using default settings with a high confidence interaction score (0.700).

Proteins were annotated and assigned to GO terms^60^ using blast2go^61^ OMICS box 3.1, InterProScan v.5.66-98.0 ^62^ and eggNOG mapper v2.1.12 ^63^ with eggNOG v5.0 ^64^ with limited terms to experimental evidence and one-to-one target orthologues prioritizing, e.g., quality. Further, the GO annotations were validated to remove all redundant terms based on the GO True Path Rule and were taxonomically filtered, using the class *Gammaproteobacteria* (Taxonomy ID:1236). Resulting GO terms were mapped to Enzyme Commission (EC) numbers.^65^ KEGG pathways were annotated by linking KEGG orthologues via eggNOG and EC numbers using OMICS box 3.1. Gene Set Enrichment Analysis for the assigned GO terms and KEGG pathways was performed using OMICS box 3.1.^66^ The enrichment parameters were used as in default settings with 1000 permutations, enrichment statistics weighted *p* = 1, detailed results for all pathways, and GO categories: Biological Process, Molecular Function, Cellular Component, Gene Set max size 500, and gene set min size 15. The results were plotted in OriginPro 2025, highlighting only sets with a false discovery rate (FDR) *q*-value < 0.05.

### Molecular Cloning

Plasmid pY359 was generated using PCR-based cloning from 3 DNA fragments: the *malK* regulatory region was amplified using primers 5′-CCT TCG CAT GCA GGA GAT GGC TTA AAT CCT C-3′ and 5′-CTT CTC CTT TAC TCA TAT GTT TGT TTT TAA TCA GGT CAA-3′ C; the *sfGFP* gene was amplified using primers 5′-CAA ACA TAT GAG TAA AGG AGG AGA AGA ACT TTT C-3′ and 5′-GCC TCT AGA TTA TTT GTA GAG CTC ATC CAT G-3′; both fragments were fused and inserted in a vector fragment from pSKA405 (unpublished) containing an rrnB T1 terminator, a p15A origin of replication, and a spectinomycin-resistance cassette.

Plasmid pY361 was generated by subcloning the *sfGFP* gene amplified using primers 5′-GAT CAT ATG AGT AAA GGA GAA GAA CTT TTC-3′ and 5′-AGT AAG CTT TTA CTT TTC GTT GGG ATC TTT C-3′ between the NdeI and *Hin*dIII restriction sites of plasmid pGDP-1 NDM-1 (Addgene plasmid #112883 ^67^). See Supporting Information for the full plasmid sequences.

### Transcriptional Reporter Assay

*E. coli* K-12 strain MG1655 carrying pY359 was grown in 5 mL 10% BHI overnight at 37 °C. Cultures were then diluted 1:100 into fresh 10% BHI and grown to OD_600_ = 0.3. Subsequently, 200 μL fractions were transferred into transparent flat-bottom polystyrene 96-well plates (Greiner), and peptides L3 (64 μg/mL), L3-K (128 μg/mL), GGH (128 μg/mL), or maltose (100 μg/mL) were added, alongside untreated controls. All media contained 50 μg/mL of spectinomycin. Cultures were incubated inside a microplate reader (Tecan Infinite 200 PRO) at 37 °C under intermittent shaking, while both optical density (600 nm) and GFP fluorescence (using an excitation wavelength of 475 nm and emission wavelength of 515 nm) were monitored in 2 min intervals for 180 min.

### Fluorescence Displacement Titrations

The DNA concentration in complexes with ethidium bromide (TCI) and DAPI (Sigma-Adrich) was at 0.6 μM of phosphates. DNA–ethidium bromide complexes were made with the dye/DNA phosphates ratio at 0.13, below that at which the binding sites become saturated.^68^ For DNA–DAPI complexes, the dye/DNA phosphates ratio was 0.015, at which the minor groove binding mode with strong fluorescence dominates.^40,41^ The DNA-dye solutions were kept overnight at 4 °C before adding peptides. Peptides and daunomycin were dissolved in Milli-Q ultrapure water to a stock concentration. All further solutions were prepared in 25 mM Tris, 5 mM NaCl buffer at pH 7.4. Increasing volumes of peptide stock were added with stirring to the DNA-dye solutions. Fluorescence was excited at 350 nm for DAPI, 540 or 545 nm for ethidium bromide for probing peptide binding, and 325 nm for ethidium bromide for probing daunomycin binding, to avoid fluorescence bleed-through. The emission was recorded using a photoluminescence spectrometer FLS 1000 (Edinburgh Instruments, UK) in quartz cuvettes. Fluorescence was recorded 10–20 min after adding each volume of peptide solution, when no further changes in the spectrum were observed.

### In Vitro Transcription/Translation Assay

Crude 12 extracts for cell-free protein synthesis were prepared as described by Kwon and Jewett^50^ Briefly, *E. coli* strain BL21­(DE3)* was grown to OD_600_ ≈ 3.0 in 2xYPTG medium at 37 °C following induction with 1 mM IPTG at OD_600_ ≈ 0.6 for the T7 RNA polymerase-based assay and without induction for the endogenous RNA polymerase-based assay. Extracts were prepared by lysing cells using sonication following resuspension at a ratio of 1:1.25 (v/v) in extraction buffer (10 mM Tris-acetate pH 8.2, 14 mM magnesium acetate, 60 mM potassium acetate, 1 mM DTT, 1× Roche Complete EDTA-free). Lysates were cleared by centrifugation (12,000*g* for 10 min). For the assay, peptides L3, L3K, or GGH (as well as a peptide-free control) were diluted to final concentrations of 1, 0.5, 0.25, and 0.1 mg/mL in 200 μL reaction volume containing final concentrations of the respective cell extract 31% (v/v), Mg­(OAC)_2_ 14 mM; ammonium hydroxide 27.4 mM; d-glutamic acid 212 mM; Hepes-KOH pH 6.5 52.5 mM; KOH 230 mM; ATP 1.1 mM; GTP 0.8 mM; CTP 0.8 mM; UTP 0.8 mM; cAMP 0.64 mM; folinic acid 68 μM; DTT 1.7 mM; creatine phosphate 63 mM; malic acid 4.4 mM; succinic acid 1.5 mM; tRNA 0.175 mg/mL; Protector RNase inhib. 8 U/mL; Roche Complete EDTA-free 1×; proteinogenic amino acids (1 mM each); serine 2 mM; glutamine 4 mM; creatine kinase 125 μg/mL.^69,70^ Reactions were started by addition of DNA template (sfGFP in pCPR-0012 for the T7 RNA polymerase-based assay and pY361 for the endogenous RNA polymerase-based assay) followed by incubation at 30 °C for 2 h. Reactions were cleared by centrifugation (16,000*g* for 5 min at 4 °C), and GFP fluorescence was determined using a microplate reader (Tecan Infinite 200 PRO) using an excitation wavelength of 475 nm and an emission wavelength of 515 nm. All measured values were normalized to those of the peptide-free control.

## Supplementary Material



## Data Availability

The mass spectra have been deposited in the PRIDE database under the project PXD068455.

## Notes

HPLC and mass spectrometry, relative quantification by proteomics, list of DEPs for L3 and L3-K with subcellular localization, DEPs belonging to the ABC transporter family, fluorescence dye displacement, and plasmid sequences and RAW data of identified and quantified proteins based on the UniProt protein database, including FC calculations and *p*-values (PDF)
