# Supplementary material for: Marine-Inspired Antimicrobial Peptides Disrupt Gene Expression at the DNA Level
Source: ACS Infect Dis. 2025 Dec 9;12(1):447–59. doi: 10.1021/acsinfecdis.5c01000 (PMC12797237; doi:10.1021/acsinfecdis.5c01000)
Supplement: Supplementary file 1 [file id5c01000_si_001.pdf]

Supporting information

Marine-inspired antimicrobial peptides disrupt gene expression at DNA level

Luisa I. Beyer, [a, b] Johannes Thoma, [a, b] Leonarda Acha Alarcon, [b, c] Ivan N. Unksov, [a] Roger Karlsson, [b, c] Juan S. Inda-Díaz,[d] and Alesia A. Tietze*[a, b]

a University of Gothenburg, Department of Chemistry and Molecular Biology, Wallenberg Centre for Molecular and Translational Medicine, Medicinaregatan 7B, 413 90 Gothenburg, Sweden, E-mail: alesia.a.tietze@gu.se

b Center for Antibiotic Resistance Research in Gothenburg, The University of Gothenburg, Box 100, 405 30 Göteborg, Sweden

c University of Gothenburg, Department of Infectious Diseases, Institute of Biomedicine, The Sahlgrenska Academy at University of Gothenburg, Box 440, 405 30 Göteborg, Sweden

d Centre for Microbiome Research, School of Biomedical Sciences, Queensland University of Technology, Translational Research Institute, Australia

Table of content

HPLC and Mass spectrometry 1
Relative quantification by proteomics 2
  Proteomic sample preparation 2
  LC-MS3 analysis 3
  Proteomic data analysis 3
  Statistical analysis 3
  Bioinformatic analysis 6
List of DEPs for L3 and L3-K with subcellular localization 8
  L3 localization of DEPs 8
  L3-K localization of DEPS 12
DEPs belonging to ABC transporter family proteins 14
Fluorescence dye displacement 16
Plasmid sequences 17
  pY359 17
  pY361 18

HPLC and Mass spectrometry

Table S1. Analytical data for synthesized peptides.

Name	Peptide	Sum formula	Calculated MW* [g/mol]	Experimental MW* [g/mol]	Δppm
**L3**	$W_DA_DV_DAWOrnTOrn_DV(Y\text{-}NO2)K]\text{-}NH2$	$C_{67}H_{98}N_{18}O_{15}$	698.3808	698.3826	2.58
**L3-K**	$W_DA_DV_DAWOrnTOrn_DV(Y\text{-}NO2)]\text{-}NH2$	$C_{61}H_{86}N_{16}O_{14}$	634.3333	634.3363	4.73

* Mass peaks detected as $[M+2H]^{2+}$

L3

(I) **(II)**

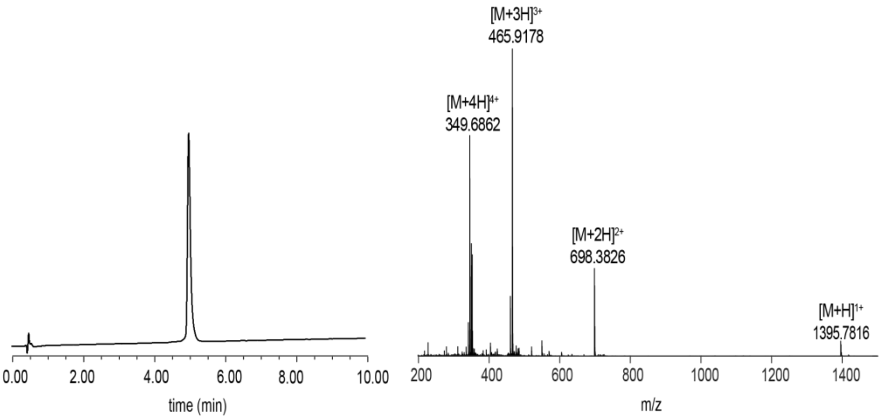

Figure S1. (I) RP-HPLC chromatogram (gradient from 10 to 50% acetonitrile in water over 10 min at 2 ml/min, detection at 214 nm) and (II) high-resolution mass spectrum for peptide **L3**.

L3-K

(I) **(II)**

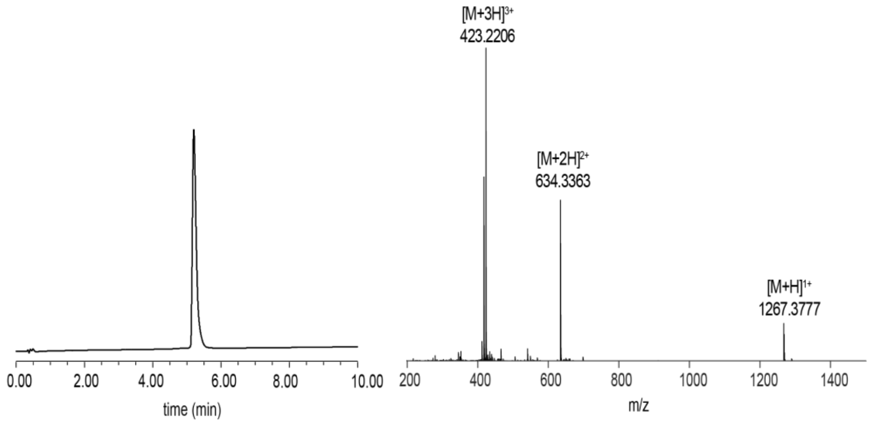

Figure S2. (I) RP-HPLC chromatogram (gradient from 10 to 50% acetonitrile in water over 10 min at 2 ml/min, detection at 214 nm) and (II) high resolution mass spectrum for peptide **L3-K**.

Relative quantification by proteomics

Proteomic sample preparation

Relative quantification was performed to compare protein expression in *E. coli* strain CCUG 31246 exposed to two antimicrobial peptides for 10 minutes compared to untreated sample (six replicates in each condition).

The sample material was homogenized by bead-beating using a FastPrep-24 instrument (MP Biomedicals) in SDS buffer, and protein concentration was determined using Pierce BCA Protein Assay Kit (Thermo Scientific) on a SpectraMax iD3 (Molecular Devices).

Samples were processed using a modified SP3 method. Samples (50 µg) were reduced in 10mM dithiothreitol at 56°C for 30min and alkylated in 20mM iodoacetamide at room temperature for 30min. Washed hydrophobic and hydrophilic Sera-Mag™ SpeedBeads (Carboxylate-Modified, Cytiva) were added to the samples with a bead to protein ratio of 10:1. Proteins were precipitated on the beads by acetonitrile (final concentration 70%), washed with 70% ethanol and dried at room temperature. Beads were resuspended in 50 µL 50 mM HEPES and proteins were digested with Trypsin/Lys-C mix [1:25, Promega] for two hours and trypsin [1:50, Promega] overnight. The peptide supernatants were collected, the magnetic beads were washed with 50 µL 50 mM HEPES and the supernatant combined with the initial peptide supernatant.

Samples were labelled using TMTpro 18-plex isobaric mass tagging reagents (Thermo Fisher Scientific). The labelled samples were pooled and purified using HiPPR Detergent Removal Resin and Pierce peptide desalting spin columns (both Thermo Scientific), according to the manufacturer´s instructions. The TMT-set was fractionated by basic reversed-phase chromatography on a Dionex Ultimate 3000 UPLC system (Thermo Fisher Scientific). Peptide separations were performed using a reversed-phase XBridge BEH C18 column (3.5 µm, 2.1x250 mm, Waters Corporation) and a stepped gradient from 3% to 54% solvent B over 70 min followed by an increase to 80% B for 3 min at a flow of 200 µL/min. Solvent A was 25mM ammonia and solvent B was 84% acetonitrile. 96 primary fractions were combined to 24 final fractions which were evaporated and reconstituted in 3% acetonitrile, 0.1% trifluoroacetic, 0.015% N-dodecyl-β-D-maltoside for LC-MS3 analysis.

LC-MS3 analysis

The fractions were analysed on an Orbitrap Eclipse™ Tribrid™ Tribrid mass spectrometer equipped with a FAIMS Pro ion mobility system and interfaced with an Easy-nLC1200 liquid chromatography system (all Thermo Fisher Scientific). Peptides were trapped on an Acclaim Pepmap 100 C18 trap column (100 µm x 2 cm, particle size 5 µm, Thermo Fisher Scientific) and separated on an in-house packed analytical column (40 cm x 75 µm, particle size 3 µm, Reprosil-Pur C18, Dr. Maisch) using a stepped gradient from 4% to 28% acetonitrile in 0.2% formic acid over 77 min at a flow of 300 nL/min. FAIMS Pro was alternating between the compensation voltages (CV) of -50 and -70, and the same data-dependent settings were used at both CVs. The precursor ion mass spectra were acquired at a resolution of 120 000 and an m/z range of 375-1500. Using a cycle time of 1.5 seconds the most abundant precursors with charges 2–7 were isolated with an m/z window of 0.7 and fragmented by collision induced dissociation (CID) at 35%. Fragment spectra were recorded in the ion trap at Rapid scan rate. Dynamic exclusion was set to 60 sec. The ten most abundant MS2 fragment ions were isolated using multi-notch isolation for further MS3 fragmentation. MS3 fragmentation was performed using higher-energy collision dissociation (HCD) at 55% and the MS3 spectra were recorded in the Orbitrap at a resolution of 50 000 and an m/z range of 100–500.

Proteomic data analysis

Raw files were processed and analyzed with Proteome Discoverer (ver 3.0, Thermo Fisher Scientific) The data was matched against UniProt *E. coli* strain K-12 database (downloaded 2023/12/06) using Sequest as a search engine with a precursor tolerance of 5 ppm and a fragment ion tolerance of 0.6 Da. Tryptic peptides were accepted with 1 missed cleavage. Methionine oxidation and protein N-terminal acetylation were set as variable modifications, cysteine carbamidomethylation, TMTpro on lysine and peptide N-termini were set as fixed modifications. Percolator was used for PSM validation with a strict FDR threshold of 1%. For quantification, TMT reporter ions were identified in the MS3 HCD spectra with 3 mmu mass tolerance and the TMT reporter intensity values for each sample were normalized on the total peptide amount. The SPS threshold was set to 65%, and a Sequest HT threshold score of 2 was chosen. Unique peptides were used for relative quantification and proteins were required to pass a protein FDR of 5%.

Statistical analysis

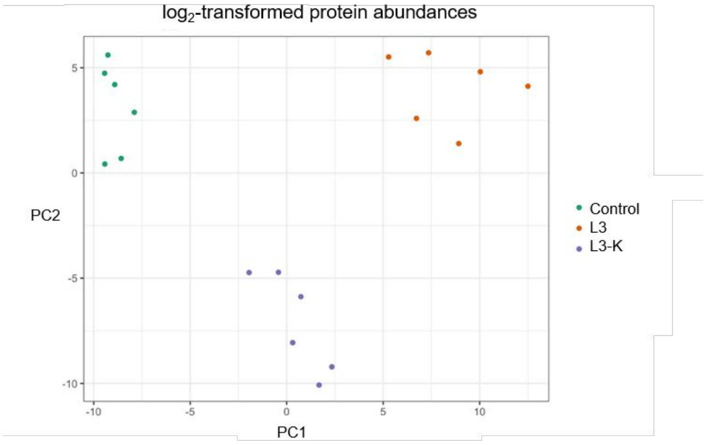

Figure S3. Sample separation along the first two principal components (PC1 and PC2) for the log_2-transformed protein abundances. The three experimental conditions were visibly split, and samples from the same experiment clustered together.

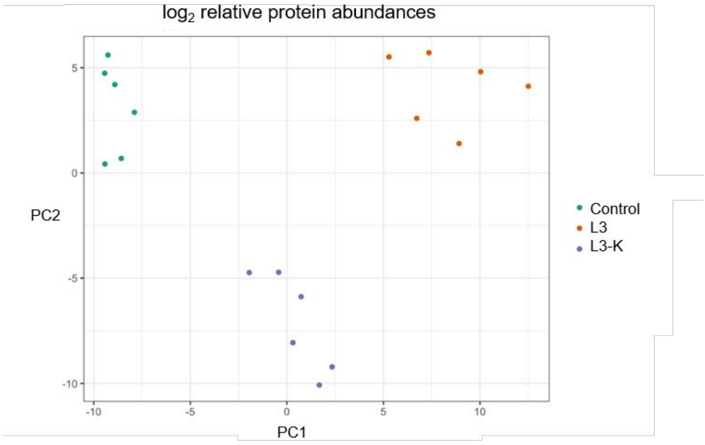

Figure S4. Sample separation along the first two principal components (PC1 and PC2) for the log_2 relative protein abundances. The three experimental conditions were visibly split, and samples from the same experiment clustered together.

Commented [JIID1]: we need to check the title (relative abundance and not log2 relative abundance), and labels (L3K and not L3K), if you can change that manually, that would be great

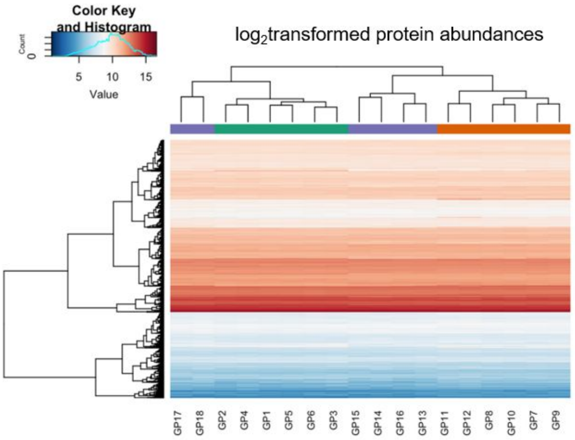

Figure S5. Heatmap of the log_2-transformed abundances shows a sample separation by experimental group, except for one sample. The green samples (GP1-GP6), the purple samples (GP13-GP18), and the red samples (GP7-GP12) correspond to control, L3, and L3-K experimental conditions.

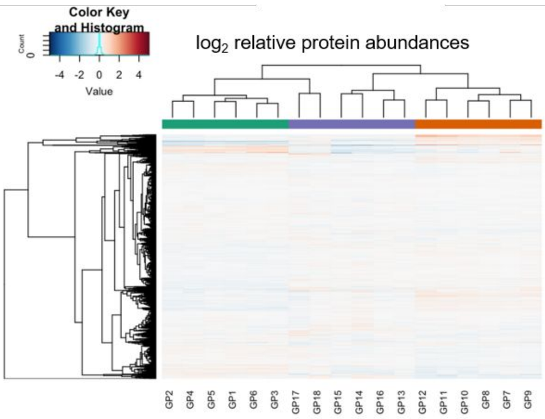

Figure S6. Heatmap of the log_2 relative abundances shows a sample separation by experimental group. The green samples (GP1-GP6), the purple samples (GP13-GP18), and the red samples (GP7-GP12) correspond to control, L3, and L3-K experimental conditions.

Bioinformatic analysis

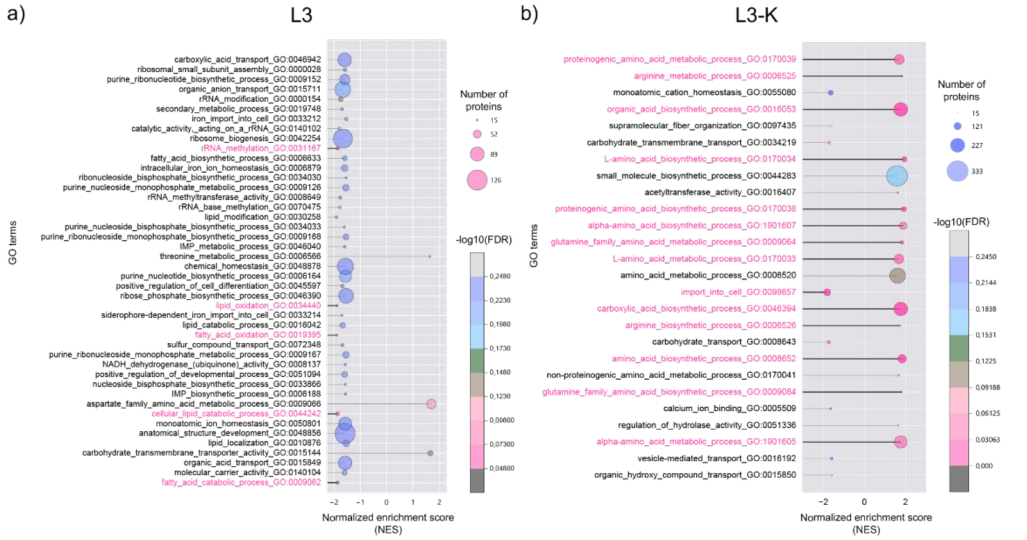

Figure S7. Gene Set Enrichment Analysis (GSEA) of GO terms. **a)** GO terms for L3; pink coloured with an FDR value of ≤ 0.05 **b)** GO terms for L3-K; pink coloured with an FDR value of ≤ 0.05.

a) GO terms L3

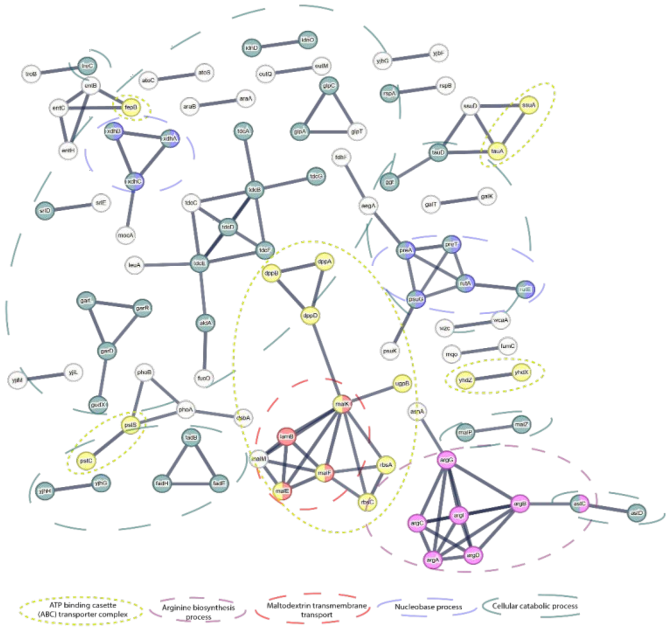

b) KEGG pathway L3

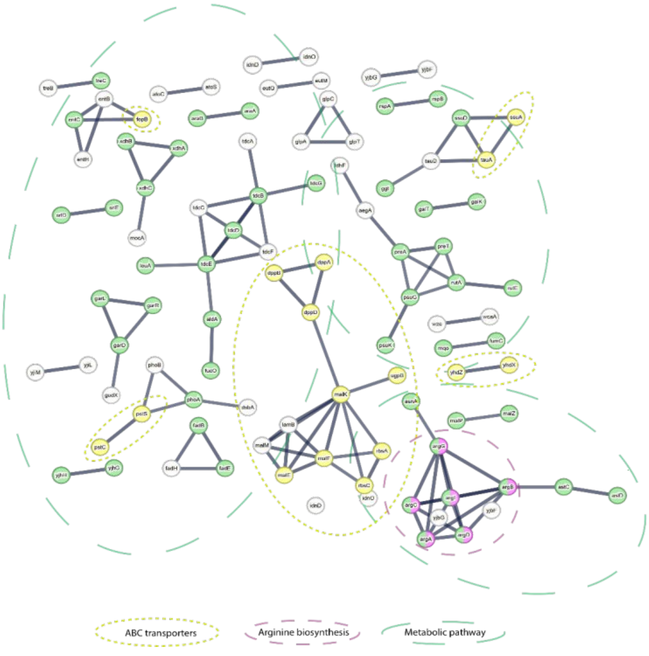

Figure S8. Protein-Protein Interaction (PPI) network of DEPs upon treatment with L3 using STRING database. a) PPI network of DEPs upon treatment with L3 grouped by different GO terms **b)** PPI network of DEPs upon treatment with L3 grouped by different KEGG pathways.

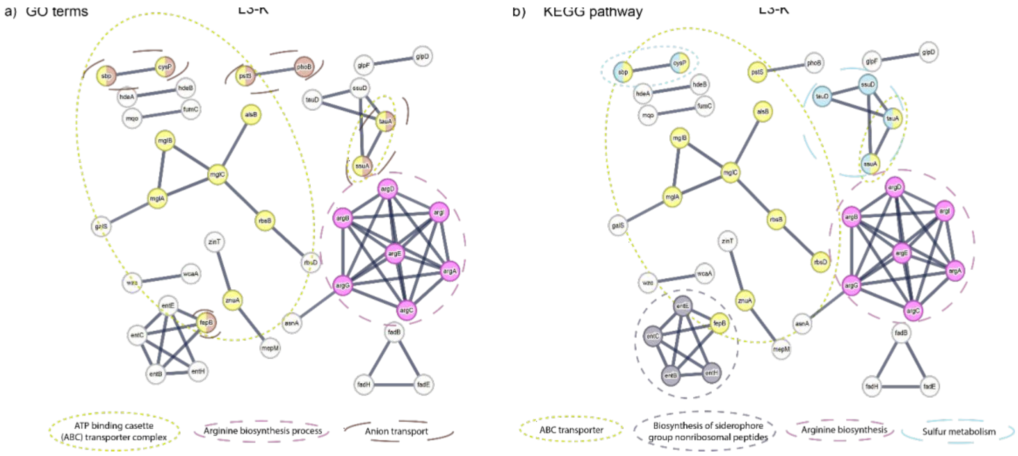

Figure S9. Protein-Protein Interaction (PPI) network of DEPs upon treatment with L3-K using STRING database. a) PPI network of DEPs upon treatment with L3-K grouped by different GO terms **b)** PPI network of DEPs upon treatment with L3-K grouped by different KEGG pathways.

List of DEPs for L3 and L3-K with subcellular localization

L3 localization of DEPs

Table S2. L3 subcellular localization of DEPs

Acession	Gene	Description	Regulated	FC	p-value
		**extracellular**			
P33343	yehD	Uncharacterized fimbrial-like protein YehD	down	-1,6	1,20E-05
P18390	yjjA	Uncharacterized protein YjjA	down	-2,5	2,85E-04
P76573	yfgI	Uncharacterized protein YfgI	down	-1,6	3,47E-03
P04949	fliC	Flagellin	up	1,8	7,92E-09
		**Outer membrane**			
P0AF56	yjcO	Sel1-repeat-containing protein YjcO	down	-1,5	2,94E-04
P02943	lamb	Maltoporin	Up	4,3	1,30E-04
P25894	loiP	Metalloprotease LoiP	Up	2,1	2,85E-06
P0AB35	YcfJ	Uncharacterized protein YcfJ	Up	2,8	1,75E-05
P32687	YjbF	Uncharacterized lipoprotein YjbF	Up	2,6	2,46E-05
P65294	YgdR	Uncharacterized lipoprotein YgdR	Up	3,3	1,33E-10
P75818	YbjP	Uncharacterized lipoprotein YbjP	Up	1,5	9,13E-07
		**Periplasm**			
P42616	yqjC	Protein YqjC	down	-3,0	4,28E-05
P77754	spy	Periplasmic chaperone Spy	down	-2,4	2,83E-04
P45766	yhdW	Putative amino-acid ABC transporter-binding protein YhdW	down	-2,4	1,16E-01
P33940	mqo	Malate:quinone oxidoreductase	down	-2,3	2,21E-06
P23857	pspE	Thiosulfate sulfurtransferase PspE	down	-2,2	1,57E-05
P0AA57	yobA	Protein YobA	down	-2,0	1,37E-04
P64506	yebY	Uncharacterized protein YebY	down	-2,0	4,55E-05
P37902	gltI	Glutamate/aspartate import solute-binding protein	down	-2,0	3,58E-04
P0AEG4	dsbA	Thiol:disulfide interchange protein DsbA	down	-1,9	3,73E-04

Q47537	tauA	Taurine-binding periplasmic protein	down	-1,8	6,36E-04
P75853	ssuA	Putative aliphatic sulfonates-binding protein	down	-1,8	2,97E-04
Q47702	yfeK	Uncharacterized protein YfeK	down	-1,8	3,09E-03
P0AG78	sbp	Sulfate-binding protein	down	-1,8	3,89E-05
P0AFL3	ppiA	Peptidyl-prolyl cis-trans isomerase A	down	-1,8	3,92E-04
P33219	yebF	Protein YebF	down	-1,8	1,81E-03
P39187	ytfJ	Uncharacterized protein YtfJ	down	-1,8	1,18E-03
P0AEU0	hisJ	Histidine-binding periplasmic protein	down	-1,8	5,91E-04
P76001	ycgJ	Uncharacterized protein YcgJ	down	-1,7	1,56E-03
P31550	thiB	Thiamine-binding periplasmic protein	down	-1,7	1,69E-04
P0AEL6	fepB	Ferric enterobactin-binding periplasmic protein FepB	down	-1,7	6,48E-05
P31133	potF	Putrescine-binding periplasmic protein PotF	down	-1,7	2,22E-04
P0AAR8	ybaV	Uncharacterized protein YbaV	down	-1,6	7,59E-03
P37329	modA	Molybdate-binding protein ModA	down	-1,6	5,68E-04
P0AEM9	tcyJ	L-cystine-binding protein TcyJ	down	-1,6	7,42E-04
P0AEQ3	glnH	Glutamine-binding periplasmic protein	down	-1,6	6,85E-04
P0C0T5	mepA	Penicillin-insensitive murein endopeptidase	down	-1,6	1,96E-03
P37028	btuF	Vitamin B12-binding protein	down	-1,6	9,81E-04
P64548	yfiR	Protein YfiR	down	-1,6	3,11E-04
P75804	yliI	Aldose sugar dehydrogenase YliI	down	-1,5	1,30E-02
P0AA99	yafK	Putative L,D-transpeptidase YafK	down	-1,5	1,50E-04
P0ABL3	napB	Periplasmic nitrate reductase, electron transfer subunit	down	-1,5	3,54E-04
P18956	ggt	Glutathione hydrolase proenzyme	down	-1,5	7,56E-06
P0AFM4	psiF	Phosphate starvation-inducible protein PsiF	down	-1,5	3,67E-03
P03841	malM	Maltose operon periplasmic protein	Up	4,8	1,65E-05
P0AG82	pstS	Phosphate-binding protein PstS	Up	4,1	8,11E-05
P0AEX9	malE	Maltose/maltodextrin-binding periplasmic protein	Up	4,0	9,04E-06
P00634	phoA	Alkaline phosphatase	Up	2,2	5,31E-04
P32688	yjbG	Uncharacterized protein YjbG	Up	1,9	1,38E-04
P0AB06	ycbK	Uncharacterized protein YcbK	Up	1,8	5,51E-06
P30860	artJ	ABC transporter arginine-binding protein 1	Up	1,6	2,60E-03
P0AG80	ugpB	sn-glycerol-3-phosphate-binding periplasmic protein UgpB	Up	1,6	1,54E-04
P23847	dppA	Dipeptide-binding protein	Up	1,5	3,24E-04
**Inner membrane**					
Q47146	fadE	Acyl-coenzyme A dehydrogenase	down	-4,3	4,53E-07
P26266	fepE	Ferric enterobactin transport protein FepE	down	-2,5	9,23E-07
P0AFX7	rseA	Anti-sigma-E factor RseA	down	-2,1	1,73E-04
P46474	yhdP	Uncharacterized protein YhdP	down	-2,0	5,93E-06
P45769	yhdZ	Uncharacterized amino-acid ABC transporter ATP-binding protein YhdZ	down	-2,0	4,31E-07
P45767	yhdX	Putative amino-acid ABC transporter permease protein YhdX	down	-1,9	7,41E-04
P69367	mdtH	Multidrug resistance protein MdtH	down	-1,8	7,48E-05
P33013	dacD	D-alanyl-D-alanine carboxypeptidase DacD	down	-1,7	3,42E-05
P0AE74	citT	Citrate/succinate antiporter	down	-1,5	2,18E-03
P02916	malF	Maltose/maltodextrin transport system permease protein MalF	Up	4,5	9,29E-05
P68187	malK	Maltose/maltodextrin import ATP-binding protein MalK	Up	4,2	2,28E-05
P0AAD8	tdcC	Threonine/serine transporter TdcC	Up	2,8	1,39E-05
P0ABB8	mgtA	Magnesium-transporting ATPase, P-type 1	Up	2,6	3,49E-10
P77414	wcaA	Putative colanic acid biosynthesis glycosyl transferase WcaA	Up	2,3	3,11E-04
P76387	wzc	Tyrosine-protein kinase wzc	Up	2,3	1,69E-05
P56580	srlE	PTS system glucitol/sorbitol-specific EIIB component	Up	2,2	2,05E-04
P0AAE8	cadB	Cadaverine/lysine antiporter	Up	2,1	7,66E-06

P0ACV2	lpxP	Lipid A biosynthesis palmitoleoyltransferase	Up	2,1	3,14E-06
P0AAG0	dppD	Dipeptide transport ATP-binding protein DppD	Up	2,0	2,15E-06
P0AEF8	dppB	Dipeptide transportsystem permease protein DppB	Up	2,0	4,32E-05
P0AFS9	mepM	Murein DD-endopeptidase MepM	Up	1,9	6,59E-07
P08194	glpT	Glycerol-3-phosphate transporter	Up	1,8	2,13E-03
P0AEP1	galP	Galactose-proton symporter	Up	1,8	4,46E-06
Q06067	atoS	Signal transduction histidine-protein kinase AtoS	Up	1,7	6,68E-04
P36672	treB	PTS system trehalose-specific EIIBC component	Up	1,7	8,79E-03
P0AFF4	nupG	Nucleoside permease NupG	Up	1,7	6,82E-06
P0A996	glpC	Anaerobic glycerol-3-phosphate dehydrogenase subunit C	Up	1,6	7,74E-04
P64545	yfgG	Protein YfgG	Up	1,6	1,60E-03
P0AGH8	pstC	Phosphate transport system permease protein PstC	Up	1,6	2,17E-07
P76359	yeeP	Putative uncharacterized protein YeeP	Up	1,6	3,20E-07
P04983	rbsA	Ribose import ATP-binding protein RbsA	Up	1,5	8,08E-03
P0AGI1	rbsC	Ribose import permease protein RbsC	Up	1,5	4,72E-03
**Cytoplasma**					
P21177	fadB	Fatty acid oxidation complex subunit alpha	down	-4,7	5,36E-05
P05042	fumC	Fumarate hydratase class II	down	-2,6	2,88E-04
P75894	rutE	Probable malonic semialdehyde reductase RutE	down	-2,4	7,23E-06
P0AEJ2	entC	Isochorismate synthase EntC	down	-2,1	2,17E-04
P24188	trhO	tRNA uridine(34) hydroxylase	down	-2,0	6,71E-08
P75898	rutA	Pyrimidine monooxygenase RutA	down	-2,0	6,01E-05
P25553	aldA	Lactaldehyde dehydrogenase	down	-2,0	9,01E-03
P37610	tauD	Alpha-ketoglutarate-dependent taurine dioxygenase	down	-1,8	1,95E-04
P80645	ssuD	Alkanesulfonate monooxygenase	down	-1,8	6,20E-04
P42593	fadH	2,4-dienoyl-CoA reductase [(2E)-enoyl-CoA-producing]	down	-1,7	2,84E-03
P0ACH1	sfsB	Sugar fermentation stimulation protein B	down	-1,6	2,65E-02
P0A8Y8	entH	Proofreading thioesterase EntH	down	-1,6	9,62E-04
P0ADI4	entB	Enterobactin synthase component B	down	-1,6	1,63E-03
P21693	dbpA	ATP-dependent RNA helicase DbpA	down	-1,6	1,96E-04
P77581	astC	Succinylornithine transaminase	down	-1,6	1,47E-04
P07658	fdhF	Formate dehydrogenase H	down	-1,6	3,35E-05
P29012	dadX	Alanine racemase, catabolic	down	-1,5	5,15E-03
P76217	astD	N-succinylglutamate 5-semialdehyde dehydrogenase	down	-1,5	3,72E-06
P33025	psuG	Pseudouridine-5'-phosphate glycosidase	up	6,3	2,29E-06
P0DPC8	ymcF	Protein YmcF	up	5,8	5,61E-05
P0A6C5	argA	Amino-acid acetyltransferase	up	5,1	5,89E-06
P39346	idnD	L-idonate 5-dehydrogenase (NAD(P)(+))	up	4,3	7,19E-07
P0AGF6	tdcB	L-threonine dehydratase catabolic TdcB	up	4,1	9,58E-04
P0ACQ7	tdcA	HTH-type transcriptional regulator TdcA	up	3,8	2,58E-05
P11868	tdcD	Propionate kinase	up	3,3	2,00E-05
P76440	preT	NAD-dependent dihydropyrimidine dehydrogenase subunit PreT	up	3,0	1,41E-05
P25889	preA	NAD-dependent dihydropyrimidine dehydrogenase subunit PreA	up	2,7	1,51E-05
P0A9P9	idnO	5-keto-D-gluconate 5-reductase	up	2,7	4,36E-06
P0AFJ5	phoB	Phosphate regulon transcriptional regulatory protein PhoB	up	2,5	5,43E-06
P0ACW6	ydcH	Uncharacterized protein YdcH	up	2,5	3,01E-04
P30235	psuK	Pseudouridine kinase	up	2,4	3,36E-05
P04391	argI	Ornithine carbamoyltransferase subunit I	up	2,4	2,79E-05
P0AGL2	tdcF	Putative reactive intermediate deaminase TdcF	up	2,4	3,96E-06
P42630	tdcG	L-serine dehydratase TdcG	up	2,3	3,05E-05
P39829	garD	Galactarate dehydratase (L-threo-forming)	up	2,2	5,70E-05
P39384	yjiM	Putative dehydratase subunit YjiM	up	2,2	2,02E-06

P0ADE2	ytfK	Uncharacterized protein YtfK	up	2,2	9,73E-06
P00963	asnA	Aspartate--ammonia ligase	up	2,1	2,44E-06
P23522	garL	5-keto-4-deoxy-D-glucarate aldolase	up	2,1	1,16E-04
P42632	tdcE	PFL-like enzyme TdcE	up	2,1	1,84E-05
P39383	yjiL	Uncharacterized protein YjiL	up	2,0	1,83E-05
P0C0L2	osmC	Peroxiredoxin OsmC	up	2,0	5,82E-06
P0A6C8	argB	Acetylglutamate kinase	up	2,0	7,98E-05
P09151	leuA	2-isopropylmalate synthase	up	2,0	1,02E-09
P28904	treC	Trehalose-6-phosphate hydrolase	up	1,9	7,07E-06
P0A9E2	soxS	Regulatory protein SoxS	up	1,9	9,94E-07
P0A9S1	fucO	Lactaldehyde reductase	up	1,9	6,35E-05
P0A853	tnaA	Tryptophanase	up	1,8	2,64E-06
P0AD53	ygaC	Uncharacterized protein YgaC	up	1,8	4,82E-06
P08202	araA	L-arabinose isomerase	up	1,8	3,22E-05
Q46801	xdhC	Putative xanthine dehydrogenase iron-sulfur-binding subunit XdhC	up	1,8	5,18E-06
P25748	galS	HTH-type transcriptional regulator GalS	up	1,8	4,69E-02
P11446	argC	N-acetyl-gamma-glutamyl-phosphate reductase	up	1,8	5,14E-04
P0ABQ2	garR	2-hydroxy-3-oxopropionate reductase	up	1,7	1,10E-04
P0A9V5	yiaG	Uncharacterized HTH-type transcriptional regulator YiaG	up	1,7	6,25E-03
P0A6T3	galK	Galactokinase	up	1,7	9,43E-04
P06993	malT	HTH-type transcriptional regulator MalT	up	1,7	1,99E-05
P0A978	cspG	Cold shock-like protein CspG	up	1,7	3,37E-04
P08204	araB	Ribulokinase	up	1,7	4,16E-05
P24169	speF	Inducible ornithine decarboxylase	up	1,7	1,73E-05
P64599	ubiT	Ubiquinone biosynthesis accessory factor UbiT	up	1,6	1,21E-05
P18335	argD	Acetylornithine/succinyldiaminopimelate aminotransferase	up	1,6	3,86E-06
P46890	ybaE	Uncharacterized protein YbaE	up	1,6	6,32E-04
Q46851	gpr	L-glyceraldehyde 3-phosphate reductase	up	1,6	1,11E-04
P69922	fucI	L-fucose isomerase	up	1,6	5,36E-05
P0A968	cspD	Cold shock-like protein CspD	up	1,6	1,59E-03
P39359	yjhH	Probable 2-dehydro-3-deoxy-D-pentonate aldolase YjhH	up	1,6	5,00E-07
Q06065	atoC	Regulatory protein AtoC	up	1,6	1,53E-05
Q46802	uacR	Putative uric acid sigma-54-dependent transcriptional regulator UacR	up	1,6	7,22E-05
P05707	srlD	Sorbitol-6-phosphate 2-dehydrogenase	up	1,6	8,81E-04
P0A6E4	argG	Argininosuccinate synthase	up	1,6	4,83E-07
P09148	galT	Galactose-1-phosphate uridylyltransferase	up	1,6	1,24E-03
P0ABF4	eutM	Bacterial microcompartment shell protein EutM	up	1,6	4,61E-08
P38105	rspB	Starvation-sensing protein RspB	up	1,6	2,86E-05
P39358	yjhG	D-xylonate dehydratase YjhG	up	1,6	9,35E-06
P25906	pdxI	Pyridoxine 4-dehydrogenase	up	1,6	8,76E-05
Q46800	xdhB	Putative xanthine dehydrogenase FAD-binding subunit XdhB	up	1,6	3,84E-06
Q46915	gudX	Glucarate dehydratase-related protein	up	1,6	5,23E-08
P37127	aegA	Putative oxidoreductase AegA	up	1,6	4,23E-06
P0A9C0	glpA	Anaerobic glycerol-3-phosphate dehydrogenase subunit A	up	1,5	1,52E-03
P23872	aes	Acetyl esterase	up	1,5	5,76E-04
P00490	malP	Maltodextrin phosphorylase	up	1,5	1,01E-05
P16681	yjdN	Protein YjdN	up	1,5	5,99E-05
P0ACQ0	rbsR	Ribose operon repressor	up	1,5	9,49E-07
P38104	rspA	Starvation-sensing protein RspA	up	1,5	5,75E-06
P37646	pdeH	Cyclic di-GMP phosphodiesterase PdeH	up	1,5	4,05E-04
P76555	eutQ	Acetate kinase EutQ	up	1,5	2,28E-03
Q46799	xdhA	Putative xanthine dehydrogenase molybdenum-binding subunit XdhA	up	1,5	1,89E-04
Q46810	mocA	Molybdenum cofactor cytidylyltransferase	up	1,5	1,47E-03
P21517	malZ	Maltodextrin glucosidase	up	1,5	2,48E-07

L3-K localization of DEPS

Table S3. L3-K subcellular localization of DEPS

Acession	Gene	Description	Regulated	FC	p-value
		**extracellular**			
P18390	yjjA	Uncharacterized protein YjjA	down	-2,9	1,54E-04
P33343	yehD	Uncharacterized fimbrial-like protein YehD	down	-2,1	4,27E-04
P76573	yfgI	Uncharacterized protein YfgI	down	-1,7	8,60E-03
		**Outer membrane**			
P25894	loiP	Metalloprotease LoiP	Up	1,6	2,43E-05
P0AB40	bhsA	Multiple stress resistance protein BhsA	Up	1,5	1,07E-03
P0AB35	YcfJ	Uncharacterized protein YcfJ	Up	2,0	1,29E-04
P32687	YjbF	Uncharacterized lipoprotein YjbF	Up	1,7	1,94E-03
P65294	YgdR	Uncharacterized lipoprotein YgdR	Up	2,7	1,51E-05
P75694	yahO	Uncharacterized protein YahO	down	-1,8	1,04E-11
P0AF56	yjcO	Sel1-repeat-containing protein YjcO	down	-1,9	5,83E-04
		**Periplasma**			
P0AG82	pstS	Phosphate-binding protein PstS	up	2,4	7,36E-03
P0AB06	ycbK	Uncharacterized protein YcbK	up	1,9	9,87E-06
P30860	artJ	ABC transporter arginine-binding protein 1	up	1,6	2,53E-02
P23857	pspE	Thiosulfate sulfurtransferase PspE	down	-5,2	1,57E-05
P0AEE5	mglB	D-galactose/methyl-galactoside binding periplasmic protein MglB	down	-4,4	2,31E-03
P64506	yebY	Uncharacterized protein YebY	down	-3,0	4,55E-05
P76001	ycgJ	Uncharacterized protein YcgJ	down	-2,8	4,28E-04
P42616	yqjC	Protein YqjC	down	-2,8	1,54E-04
P0AAR8	ybaV	Uncharacterized protein YbaV	down	-2,5	8,59E-03
P02925	rbsB	Ribose import binding protein RbsB	down	-2,4	2,56E-04
P33219	yebF	Protein YebF	down	-2,4	2,02E-05
P64548	yfiR	Protein YfiR	down	-2,4	1,12E-06
Q47702	yfeK	Uncharacterized protein YfeK	down	-2,3	1,47E-04
P39187	ytfJ	Uncharacterized protein YtfJ	down	-2,2	3,91E-04
P0AA57	yobA	Protein YobA	down	-2,2	1,37E-04
P37329	modA	Molybdate-binding protein ModA	down	-2,2	1,10E-05
P0AEL6	fepB	Ferric enterobactin-binding periplasmic protein FepB	down	-2,1	7,32E-07
P0AFL3	ppiA	Peptidyl-prolyl cis-trans isomerase A	down	-2,1	2,13E-05
P0AEG4	dsbA	Thiol:disulfide interchange protein DsbA	down	-2,1	1,44E-04
P31550	thiB	Thiamine-binding periplasmic protein	down	-2,1	2,34E-05
P33940	mqo	Malate:quinone oxidoreductase	down	-2,0	2,21E-06
P37028	btuF	Vitamin B12-binding protein	down	-2,0	1,35E-04
P0ADA1	tesA	Thioesterase 1/protease 1/lysophospholipase L1	down	-2,0	8,41E-05
P0AET2	hdeB	Acid stress chaperone HdeB	down	-1,9	4,00E-05
P0AEQ3	glnH	Glutamine-binding periplasmic protein	down	-1,9	4,93E-04
P75853	ssuA	Putative aliphatic sulfonates-binding protein	down	-1,9	2,83E-03
P0AEU0	hisJ	Histidine-binding periplasmic protein	down	-1,9	1,47E-03
P0AEM9	tcyJ	L-cystine-binding protein TcyJ	down	-1,8	4,68E-04
P0ABL3	napB	Periplasmic nitrate reductase, electron transfer subunit	down	-1,8	2,20E-05
P39265	alsB	D-allose-binding periplasmic protein	down	-1,8	2,41E-05

P39325	ytfQ	Galactofuranose-binding protein YtfQ	down	-1,8	5,78E-04
P37902	gltI	Glutamate/aspartate import solute-binding protein	down	-1,8	3,58E-04
P16700	cysP	Thiosulfate-binding protein	down	-1,7	1,48E-03
P0ADS9	yggN	Uncharacterized protein YggN	down	-1,7	1,44E-04
P77754	spy	Periplasmic chaperone Spy	down	-1,7	1,52E-04
P39172	znuA	High-affinity zinc uptake system protein ZnuA	down	-1,7	4,27E-04
P15028	fecB	Fe(3+) dicitrate-binding periplasmic protein FecB	down	-1,7	1,75E-04
Q47537	tauA	Taurine-binding periplasmic protein	down	-1,7	5,66E-03
P76108	ydcS	Bifunctional polyhydroxybutyrate synthase / ABC transporter periplasmic binding protein	down	-1,7	3,51E-04
P0ADU5	ygiW	Protein YgiW	down	-1,7	6,89E-04
P30859	artI	Putative ABC transporter arginine-binding protein 2	down	-1,6	7,48E-04
P0AB24	efeO	Iron uptake system component EfeO	down	-1,6	1,75E-04
P0AGD1	sodC	Superoxide dismutase [Cu-Zn]	down	-1,6	4,90E-03
P76344	zinT	Metal-binding protein ZinT	down	-1,6	1,22E-02
P75804	yliI	Aldose sugar dehydrogenase YliI	down	-1,6	6,17E-04
P0C0T5	mepA	Penicillin-insensitive murein endopeptidase	down	-1,5	7,41E-04
P0AFM4	psiF	Phosphate starvation-inducible protein PsiF	down	-1,5	7,03E-03
P0AG78	sbp	Sulfate-binding protein	down	-1,5	8,47E-03
P0AES9	hdeA	Acid stress chaperone HdeA	down	-1,5	1,42E-03
P76076	ydbL	Uncharacterized protein YdbL	down	-1,5	9,01E-05
**Inner membrane**					
P0ABB8	mgtA	Magnesium-transporting ATPase, P-type 1	Up	2,8	4,23E-07
P0AFS9	mepM	Murein DD-endopeptidase MepM	Up	2,1	8,29E-08
P0AAE8	cadB	Cadaverine/lysine antiporter	Up	2,1	9,88E-06
P77414	wcaA	Putative colanic acid biosynthesis glycosyl transferase WcaA	Up	2,0	2,95E-03
P0ACV2	lpxP	Lipid A biosynthesis palmitoleoyltransferase	Up	1,9	5,21E-05
P76387	wzc	Tyrosine-protein kinase wzc	Up	1,9	1,82E-03
P27242	waaU	Lipopolysaccharide 1,2-N-acetylglucosaminetransferase	Up	1,6	4,32E-02
P0A8D9	yfbV	UPF0208 membrane protein YfbV	Up	1,5	7,64E-04
P75829	ybjX	Uncharacterized protein YbjX	Up	1,5	2,29E-04
P23200	mglC	Galactose/methyl galactoside import permease protein MglC	down	-3,2	9,72E-03
P0AAG8	mglA	Galactose/methyl galactoside import ATP-binding protein MglA	down	-3,0	1,03E-02
P0AER0	glpF	Glycerol uptake facilitator protein	down	-2,4	2,53E-02
P26266	fepE	Ferric enterobactin transport protein FepE	down	-2,0	1,02E-04
P0A830	dctA	Aerobic C4-dicarboxylate transport protein	down	-2,0	2,08E-02
P42619	yqjF	Inner membrane protein YqjF	down	-1,9	3,36E-06
Q47146	fadE	Uncharacterized protein YhdP	down	-1,9	4,03E-05
P46474	yhdP	D-alanyl-D-alanine carboxypeptidase DacD	down	-1,8	2,34E-04
P33013	dacD	Galactose/methyl galactoside import permease protein MglC	down	-1,6	9,72E-03
P36672	treB	PTS system trehalose-specific EIIBC component	down	-1,6	3,35E-02
**Cytoplasma**					
P25748	galS	HTH-type transcriptional regulator GalS	down	-4,0	0,003143
P21177	fadB	Fatty acid oxidation complex subunit alpha	down	-2,6	0,00081
P39361	sgcR	Putative sgc region transcriptional regulator	down	-2,5	0,046511

P24188	trhO	tRNA uridine(34) hydroxylase	down	-2,1	5,55E-08
P05042	fumC	Fumarate hydratase class II	down	-2,0	0,000926
P37610	tauD	Alpha-ketoglutarate-dependent taurine dioxygenase	down	-2,0	8,77E-05
P0AEJ2	entC	Isochorismate synthase EntC	down	-2,0	8,84E-05
P13035	glpD	Aerobic glycerol-3-phosphate dehydrogenase	down	-1,9	0,000373
P0A8Y8	entH	Proofreading thioesterase EntH	down	-1,8	0,000286
P0ADI4	entB	Enterobactin synthase component B	down	-1,7	0,000469
P10378	entE	Enterobactin synthase component E	down	-1,7	0,000344
P77171	ydcI	Uncharacterized HTH-type transcriptional regulator YdcI	down	-1,7	1,28E-05
P21693	dbpA	ATP-dependent RNA helicase DbpA	down	-1,6	6,66E-05
P80645	ssuD	Alkanesulfonate monooxygenase	down	-1,6	0,000485
P39360	yjhI	Uncharacterized HTH-type transcriptional regulator YjhI	down	-1,6	0,000109
P25553	aldA	Lactaldehyde dehydrogenase	down	-1,6	0,049233
P29012	dadX	Alanine racemase, catabolic	down	-1,6	0,005025
P04982	rbsD	D-ribose pyranase	down	-1,6	0,011363
P0ADX7	yhhA	Uncharacterized protein YhhA	down	-1,6	0,002358
P42593	fadH	2,4-dienoyl-CoA reductase [(2E)-enoyl-CoA-producing]	down	-1,5	0,012321
P0A6C5	argA	Amino-acid acetyltransferase	up	5,4	2,87E-05
P0DPC8	ymcF	Protein YmcF	up	5,2	0,000173
P04391	argI	Ornithine carbamoyltransferase subunit I	up	2,5	4,09E-08
P0A6C8	argB	Acetylglutamate kinase	up	2,2	1,94E-06
P11446	argC	N-acetyl-gamma-glutamyl-phosphate reductase	up	2,1	1,93E-06
P0A9V5	yiaG	Uncharacterized HTH-type transcriptional regulator YiaG	up	1,9	1,57E-06
P0AFJ5	phoB	Phosphate regulon transcriptional regulatory protein PhoB	up	1,8	3,93E-05
P0A978	cspG	Cold shock-like protein CspG	up	1,8	6,02E-07
P0A853	tnaA	Tryptophanase	up	1,8	2,56E-09
P0ADE2	ytfK	Uncharacterized protein YtfK	up	1,8	0,000917
P18335	argD	Acetylornithine/succinyldiaminopimelate aminotransferase	up	1,7	8,13E-06
P0C0L2	osmC	Peroxiredoxin OsmC	up	1,7	7,86E-06
P0A6E4	argG	Argininosuccinate synthase	up	1,7	7,27E-05
P00963	asnA	Aspartate--ammonia ligase	up	1,7	8,78E-05
P0AD53	ygaC	Uncharacterized protein YgaC	up	1,6	1,82E-05
P37127	aegA	Putative oxidoreductase AegA	up	1,6	2,87E-07
P23908	argE	Acetylornithine deacetylase	up	1,6	1,78E-05
P64599	ubiT	Ubiquinone biosynthesis accessory factor UbiT	up	1,6	6,35E-06
P0ADH5	fimB	Type 1 fimbriae regulatory protein FimB	up	1,6	0,000412
P09151	leuA	2-isopropylmalate synthase	up	1,5	4,15E-07
P16681	yjdN	Protein YjdN	up	1,5	0,000512

DEPs belonging to ABC transporter family proteins

Table S4. ABC transporter proteins differentially expressed after treatment with L3

**Transporter**	**gene**	**Substrate**	**Biological function**	**regulation**	**Log2 FC**	**p-value**
Mal	malF*	Maltose (malto-oligosaccharides prediced), transmembrane protein	Disaccharaide importer	Up	4.5	9.29E-05
Mal	malK	Maltose (malto-oligosaccharides prediced), ATP-binding protein	Disaccharaide importer	up	4.2	2.28E-05
Pst	pstS*	Phosphate; solute-binding protein	High-affinity phosphate transport	Up	4.1	8.11E-05
Dpp	dppB	Dipeptide, 5-aminolevulinic acid (ALA), transmembrane protein	Dipeptide transporter	up	2.0	4.32E-05

Dpp	dppD	Dipeptide, 5-aminolevulinic acid (ALA); ATP-binding protein	Dipeptide transporter	up	2.0	2.15E-06
Ugp	ugpB	Sn-glycerol 3-phosphate, solute-binding protein	Glycerol-phosphate transporter protein	up	1.6	1.54E-04
Pst	pstC	Phosphate, binding-protein-dependent transport system	High-affinity phosphate transport	Up	1.6	2.17E-07
Dpp	dppA	Dipeptide, 5-aminolevulinic acid (ALA), solute-binding protein	Dipeptide transporter	up	1.5	3.24E-04
Rbs	rbsA	D-ribose; ATP-binding protein	Monosaccharide importer	up	1.5	8.08E-03
Yhd	yhdZ	unknown	Putative polar amino acid transporter	Down	-2.0	4.31E-07
Yhd	yhdX	unknown	Putative polar amino acid transporter	Down	-1.9	7.41E-04
Ssu	ssuA	Sulfonate, solute-binding protein	Aliphatic sulfonate transporter	down	-1.8	2.97E-04
Tau	tauA	Taurine, solute-binding protein	Taurine porter	down	-1.8	6.36E-04
Fep	fepB*	Ferrientero-bactin, solute-binding protein	Iron porter	down	-1.7	6.48E-05

* reported as DNA damage response gene based on Khil et al., 2002

Table S5. ABC transporter proteins differentially expressed after treatment with L3-K

Transporter	gene	Substrate	Biological function	regulation	Log2 FC	p-value
Mgl	mglB	β-D -galactose	Monosaccharide porter, solute binding protein	Down	-4.4	2.31E-03
Mgl	mglC*	β-D -galactose	Monosaccharide porter, transmembrane protein	Down	-3.2	9.72E-03
Mgl	mglA*	β-D -galactose	Monosaccharide porter, ATP-binding protein	Down	-3.0	1.03E-02
Rbs	rbsB	D-ribose	Monosaccharide importer; solute-binding protein	down	-2.4	2.56E-04
fep	fepB*	Ferrientero-bactin	Iron porter; solute-bindig protein	down	-2.1	7.32E-07
Ssu	ssuA	Sulfonate	Aliphatic sulfonate transporter; solute-binding protein	down	-1.9	2.83E-03
Als	alsB	Allose, ribose	Monosaccharide importer, solute-binding protein	down	-1.8	2.41E-05
Cys	cysP	Thiosulfate	Sulfate/Thiosulfate importer, solute-binding protein	down	-1.7	1.48E-03
Tau	TauA	Taurine	Taurine porter, solute-binding protein	down	-1.7	1.48E-03
Znu	znuA	Zn2+	High-affinity zinc uptake solute-binding protein	down	-1.7	4.27E-04
Spb	Sbp	Sulfate, thiosulfate	Sulfate/thiosulfate porter. TMD/NBD components from the Cys sytem, sulfate binding protein, periplasmic binding protein	down	-1.5	8.47E-03
Pst	pstS*	Phosphate	High-affinity phosphate transport, solute-binding protein	up	2.4	7.36E-03

* reported as DNA damage response gene based on Khil et al., 2002

Fluorescence dye displacement

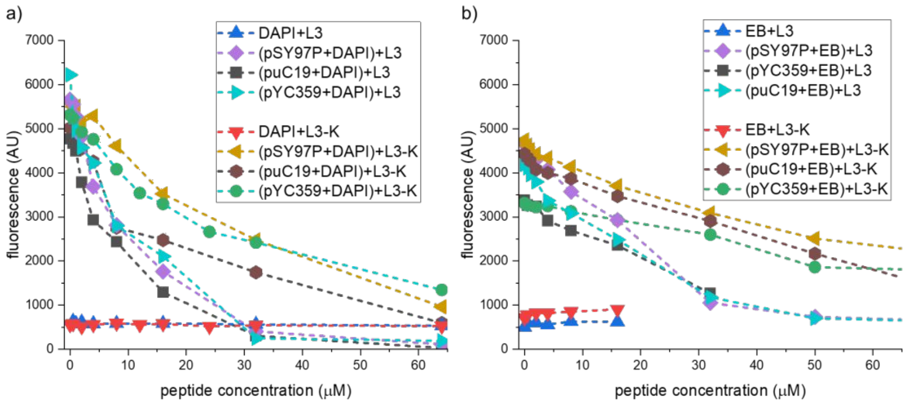

Figure S9. Fluorescence of (a) DAPI and (b) ethidium bromide, free and bound to plasmid DNA puC19, pSY97P, and pYC359, in presence of increasing concentrations of L3 and L3-K peptides.

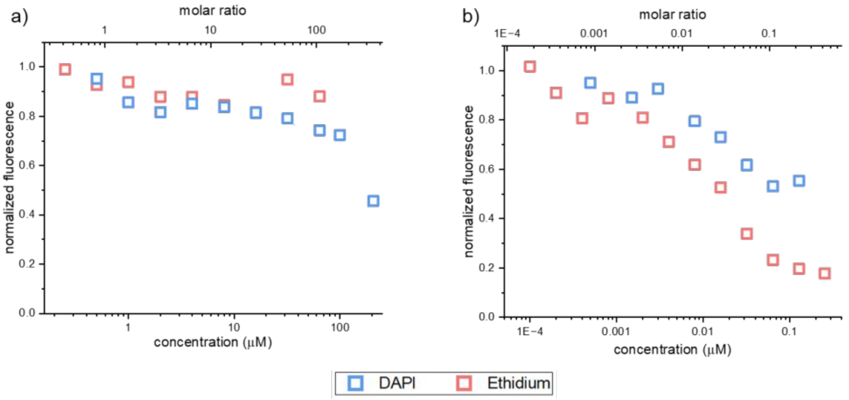

Figure S10. Benchmark controls for the dye displacement assay on plasmid pYC359. Fluorescence of DNA-bound DAPI and EtBr in the presence of increasing concentrations of (a) GGH ATCUN peptide and (b) daunomycin. Peak fluorescence (460 nm for DAPI, 610 nm for EtBr) is normalized to the pre-addition signal. Molar ratio is ligand (peptide or daunomycin) to DNA phosphates (0.6 μM).

(a)

(b)

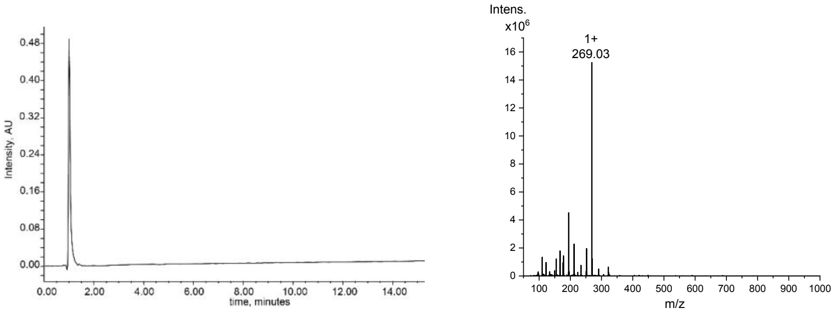

Figure S11. (a) RP-HPLC chromatogram (detection at 214 nm) and (b) high resolution mass spectrum of purified GGH ATCUN peptide. HPLC gradient: 0 – 15% acetonitrile in water in 15 minutes total.

Plasmid sequences

pY359

ggcatcaaataaaacgaaaggctcagtcgaaagactgggcctttcgttttatctgttgtttgtcggtgaacgctctcctgagtaggacaaatccgccgccctagacctag
gggatatattccgcttcctcgctcactgactcgctacgctcggtcgttcgactgcggcgagcggaaatggcttacgaacggggcggagatttcctggaagatgccagga
agatacttaacagggaagtgagagggccgcggcaaagccgtttttccataggctccgcccccctgacaagcatcacgaaatctgacgctcaaatcagtggtggcga
aacccgacaggactataaagataccaggcgtttccccctggcggctccctcgtgcgctctcctgttcctgcctttcggtttaccggtgtcattccgctgttatggccgcgtttg
tctcattccacgcctgacactcagttccgggtaggcagttcgctccaagctggactgtatgcacgaaccccccgttcagtccgaccgctgcgccttatccggtaactatcg
tcttgagtccaacccggaaagacatgcaaaagcaccactggcagcagccactggtaattgatttagaggagttagtcttgaagtcatgcgccggttaaggctaaactg
aaaggacaagttttggtgactgcgctcctccaagccagttacctcggttcaaagagttggtagctcagagaaccttcgaaaaaccgccctgcaaggcggttttttcgtttt
cagagcaagagattacgcgcagaccaaaacgatctcaagaagatcatcttattaatcagataaaatattactagatttcagtgcaatttatctcttcaaatgtagcacctg
aagtcagccccatacgatataagttgttactagtgcttggattctcaccaataaaaaacgcccggcggcaaccgagcgttctgaacaaatccagatggagttctgaggt
cattactggatctatcaacaggagtccaagcgagctcttatttgccgactaccttggtgatctcgcctttcacgtagtggacaaattcttccaactgatctgcgcgcgaggc
caagcgatcttcttcttgtccaagataagcctgtctagcttcaagtatgacgggctgatactgggccggcaggcgctccattgcccagtcggcagcgacatccttcggcg
cgattttgccggttactgcgctgtaccaaatgcgggacaacgtaagcactacatttcgctcatcgccagcccagtcgggcggcgagttccatagcgttaaggtttcattta
gcgcctcaaatagatcctgttcaggaaccggatcaaagagttcctccgccgctggacctaccaaggcaacgctatgttctcttgcttttgtcagcaagatagccagatca
atgtcgatcgtggctggctcgaagatacctgcaagaatgtcattgcgctgccattctccaaattgcagttcgcgcttagctggataacgccacggaatgatgtcgtcgtgc
acaacaatggtgacttctacagcgcggagaatctcgctctctccaggggaagccgaagtttccaaaaggtcgttgatcaaagctcgccgcgttgtttcatcaagccttac
ggtcaccgtaaccagcaaatcaatatcactgtgtggcttcaggccgccatccactgcggagccgtacaaatgtacggccagcaacgtcggttcgagatggcgctcga
tgacgccaactacctctgatagttgagtcgatacttcggcgatcaccgcttccctcatgcgaaacgatcctcatcctgtctcttgatcagatattgatcccctgcgccatcag
atccttggcggcaagaaagccatccagtttactttgcagggcttcccaaccttaccagagggcgccccagctggcaattccgacgtccaaaaaacccctcaagaccc
gtttagaggccccaaggggttatgctagttaatttttaaagtatgggcaatcaattgctcctgttaaaattgctttagaaatactttggcagcggtttgttgtattgagtttcatttg
cgcattggttaaatggaaagtgacagtacgctcactgcaacctaatatttttgaaatatcccaagagctttttccttcgcatgcaggagatggcttaaatcctccacccccctg
gctttttttatgggggaggaggcgggaggatgagaacacggcttctgtgaactaaaccgaggtcatgtaaggaatttcgtgatgttgcttgcaaaaatcgtggcgattttat
gtgcgcatctccacattaccgccaattctgtaacagagatcacacaaagcgacggtggggcgtaggggcaaggaggatggaaagaggttgccgtataaagaaact
agagtccgtttaggtgttttcacgagcacttcaccaacaaggaccatagattatgaaataaaaacaggtgcacgcatcctcgcattatccgcattaacgacgatgatgt
tttccgcctcggctctcgccaaaatcgaagaaggtaaactggtaatctggattaacggcgataaaggctataacggtctcgctgaagtcggtaagaaattcgagaaag
ataccggaattaaagtcaccgttgagcatccggataaactggaagagaaattcccacaggttgcggcaactggcgatggccctgacattatcttctgggcacacgac
cgctttggtggctacgctcaatctggcctgttggctgaaatcaccccggacaaagcgttccaggacaagctgtatccgtttacctgggatgccgtacgttacaacggcaa
gctgattgcttacccgatcgctgttgaagcgttatcgctgatttataacaaagatctgctgccgaacccgccaaaaacctgggaagagatcccggcgctggataaaga
actgaaagcgaaaggtaagagcgcgctgatgttcaacctgcaagaaccgtacttcacctggccgctgattgctgctgacggggttatgcgttcaagtatgaaaacgg
caagtacgacattaaagacgtgggcgtggataacgctggcgcgaaagcgggtctgaccttcctggttgacctgattaaaaacaaacatatgagtaaaggagaagaa
cttttcactggagttgtcccaattcttgttgaattagatggtgatgttaatgggcacaaattttctgtccgtggagagggtgaaggtgatgctacaaacggaaaactcaccctt
aaatttatttgcactactggaaaactacctgttccgtggccaacacttgtcactactctgacctatggtgttcaatgcttttcccgttatccggatcacatgaaacggcatgact
ttttcaagagtgccatgcccgaaggttatgtacaggaacgcactatatctttcaaagatgacgggacctacaagacgcgtgctgaagtcaagtttgaaggtgatacccttgttaatcgtatcgagttaaagggtattgattttaaagaagatggaaacattcttggacacaaactcgagtacaactttaactcacacaatgtatacatcacggcagacaa
acaaaagaatggaatcaaagctaacttcaaaattcgccacaacgttgaagatggttccgttcaactagcagaccattatcaacaaaatactccaattggcgatggccc
tgtccttttaccagacaaccattacctgtcgacacaatctgtcctttcgaaagatcccaacgaaaagcgtgaccacatggtccttcttgagtttgtaactgctgctgggatta
cacatggcatggatgagctctacaaataatctaga

pY361
agtaaggcaaccccgccagcctagccgggagatctggcctattggttaaaaaatgagctgatttaacaaaaatttaacgcgaattttaacaaaatattaacgcttacaa
tttaggtggcacttttcggggaaatgtgcgcggaacccctatttgtttatttttctaaatacattcaaatatgtatccgctcatgagacaataaccctgataaatgcttctctaga
aataattttgtttaactttaagaaggagatatcatatgagtaaaggagaagaacttttcactggagttgtcccaattcttgttgaattagatggtgatgttaatgggcacaaatt
ttctgtccgtggagagggtgaaggtgatgctacaaacggaaaactcacccttaaatttatttgcactactggaaaactacctgttccgtggccaacacttgtcactactctg
acctatggtgttcaatgcttttcccgttatccggatcacatgaaacggcatgactttttcaagagtgccatgcccgaaggttatgtacaggaacgcactatatctttcaaaga
tgacgggacctacaagacgcgtgctgaagtcaagtttgaaggtgatacccttgttaatcgtatcgagttaaagggtattgattttaaagaagatggaaacattcttggaca
caaactcgagtacaactttaactcacacaatgtatacatcacggcagacaaacaaaagaatggaatcaaagctaacttcaaaattcgccacaacgttgaagatggtt
ccgttcaactagcagaccattatcaacaaaatactccaattggcgatggccctgtccttttaccagacaaccattacctgtcgacacaatctgtcctttcgaaagatccca
acgaaaagcgtgaccacatggtccttcttgagtttgtaactgctgctgggattacacatggcatggatgagctctacaaataaaagcttgcggccgcactcgagcacca
ccaccaccaccactgagatccggctgctaacaaagcccgaaaggaagctgagttggctgctgccaccgctgagcaaaaggccatccgtcaggatggccttctggtg
gcacttttcggggaaatgtgcgcggaacccctatttgtttatttttctaaatacattcaaatatgtatccgctcatgaattaattcttagaaaaactcatcgagcatcaaatgaa
actgcaatttattcatatcaggattatcaataccatatttttgaaaaagccgtttctgtaatgaaggagaaaactcaccgaggcagttccataggatggcaagatcctggta
tcggtctgcgattccgactcgtccaacatcaatacaacctattaatttcccctcgtcaaaaataaggttatcaagtgagaaatcaccatgagtgacgactgaatccggtg
agaatggcaaaagtttatgcatttctttccagacttgttcaacaggccagccattacgctcgtcatcaaaatcactcgcatcaaccaaaccgttattcattcgtgattgcgcc
tgagcgagacgaaatacgcgatcgctgttaaaaggacaattacaaacaggaatcgaatgcaaccggcgcaggaacactgccagcgcatcaacaatattttcacct
gaatcaggatattcttctaatacctggaatgctgttttcccggggatcgcagtggtgagtaaccatgcatcatcaggagtacggataaaatgcttgatggtcggaagagg
cataaattccgtcagccagtttagtctgaccatctcatctgtaacatcattggcaacgctacctttgccatgtttcagaaacaactctggcgcatcgggcttcccatacaatc
gatagattgtcgcacctgattgcccgacattatcgcgagcccatttatacccatataaatcagcatccatgttggaatttaatcgcggcctagagcaagacgtttcccgttg
aatatggctcataacaccccttgtattactgtttatgtaagcagacagttttattgttcatgaccaaaatcccttaacgtgagttttcgttccactgagcgtcagaccccgtaga
aaagatcaaaggatcttcttgagatcctttttttctgcgcgtaatctgctgcttgcaaacaaaaaaaccaccgctaccagcggtggtttgtttgccggatcaagagctacca
actctttttccgaaggtaactggcttcagcagagcgcagataccaaatactgtccttctagtgtagccgtagttaggccaccacttcaagaactctgtagcaccgcctaca
tacctcgctctgctaatcctgttaccagtggctgctgccagtggcgataagtcgtgtcttaccgggttggactcaagacgatagttaccggataaggcgcagcggtcggg
ctgaacggggggttcgtgcacacagcccagcttggagcgaacgacctacaccgaactgagatacctacagcgtgagctatgagaaagcgccacgcttcccgaag
ggagaaaggcggacaggtatccggtaagcggcagggtcggaacaggagagcgcacgagggagcttccagggggaaacgcctggtatctttatagtcctgtcggg
tttcgccacctctgacttgagcgtcgatttttgtgatgctcgtcaggggggcggagcctatggaaaaacgccagcaacgcggcctttttacggttcctggccttttgctggcc
ttttgctcacatgttctttcctgcgttatcccctgattctgtggataaccgtattaccgcctttgagtgagctgataccgctcgccgcagccgaacgaccgagcgcagcgagt
cagtgagcgaggaagcggaagagcgcctgatgcggtattttctccttacgcatctgtgcggtatttcacaccgcaatggtgcactctcagtacaatctgctctgatgccgc
atagttaagccagtatacactccgctatcgctacgtgactgggtcatggctgcgccccgacacccgccaacacccgctgacgcgccctgacgggcttgtctgctcccg
gcatccgcttacagacaagctgtgaccgtctccgggagctgcatgtgtcagaggttttcaccgtcatcaccgaaacgcgcgaggcagctgcggtaaagctcatcagc
gtggtcgtgaagcgattcacagatgtctgcctgttcatccgcgtccagctcgttgagtttctccagaagcgttaatgtctggcttctgataaagcgggccatgttaagggcg
gtttttcctgtttggtcactgatgcctccgtgtaagggggatttctgttcatgggggtaatgataccgatgaaacgagagaggatgctcacgatacgggttactgatgatga
acatgcccggttactggaacgttgtgagggtaaacaactggcggtatggatgcggcgggaccagagaaaaatcactcagggtcaatgccagcgcttcgttaataca
gatgtaggtgttccacagggtagccagcagcatcctgcgatgcagatccggaacataatggtgcagggcgctgacttccgcgtttccagactttacgaaacacggaa
accgaagaccattcatgttgttgctcaggtcgcagacgttttgcagcagcagtcgcttcacgttcgctcgcgtatcggtgattcattctgctaacc
